# Map-based Cloning and Characterization of the *BPH18* Gene from Wild Rice Conferring Resistance to Brown Planthopper (BPH) Insect Pest

**DOI:** 10.1038/srep34376

**Published:** 2016-09-29

**Authors:** Hyeonso Ji, Sung-Ryul Kim, Yul-Ho Kim, Jung-Pil Suh, Hyang-Mi Park, Nese Sreenivasulu, Gopal Misra, Suk-Man Kim, Sherry Lou Hechanova, Hakbum Kim, Gang-Seob Lee, Ung-Han Yoon, Tae-Ho Kim, Hyemin Lim, Suk-Chul Suh, Jungil Yang, Gynheung An, Kshirod K. Jena

**Affiliations:** 1 Department of Agricultural Biotechnology, National Institute of Agricultural Sciences, Jeonju, Korea; 2 Plant Breeding Division, International Rice Research Institute (IRRI), Metro Manila, Philippines; 3 National Institute of Crop Science, Suwon, Korea; 4 IRRI-Korea Office, National Institute of Crop Science, Rural Development Administration, Jeonju, Korea; 5 Division of Tree Breeding, National Institute of Forest Science Institute, Suwon, Korea; 6 Department of Plant Molecular Systems Biotechnology and Crop Biotech Institute, Kyung Hee University, Yongin, Korea

## Abstract

Brown planthopper (BPH) is a phloem sap-sucking insect pest of rice which causes severe yield loss. We cloned the *BPH18* gene from the BPH-resistant introgression line derived from the wild rice species *Oryza australiensis*. Map-based cloning and complementation test revealed that the *BPH18* encodes CC-NBS-NBS-LRR protein. BPH18 has two NBS domains, unlike the typical NBS-LRR proteins. The *BPH18* promoter::*GUS* transgenic plants exhibited strong GUS expression in the vascular bundles of the leaf sheath, especially in phloem cells where the BPH attacks. The BPH18 proteins were widely localized to the endo-membranes in a cell, including the endoplasmic reticulum, Golgi apparatus, *trans*-Golgi network, and prevacuolar compartments, suggesting that BPH18 may recognize the BPH invasion at endo-membranes in phloem cells. Whole genome sequencing of the near-isogenic lines (NILs), NIL-*BPH18* and NIL-*BPH26*, revealed that *BPH18* located at the same locus of *BPH26*. However, these two genes have remarkable sequence differences and the independent NILs showed differential BPH resistance with different expression patterns of plant defense-related genes, indicating that *BPH18* and *BPH26* are functionally different alleles. These findings would facilitate elucidation of the molecular mechanism of BPH resistance and the identified novel alleles to fast track breeding BPH resistant rice cultivars.

The brown planthopper (BPH), *Nilaparvata lugens* Stål (Homoptera:Delphacidae) is the most destructive insect pest affecting rice plants in many rice growing countries besides rice stem borers that affect rice production in some regions under favorable climatic conditions. It is a phloem sap–sucking insect, making it a vector for the transmission of viral diseases such as ragged stunt and grassy stunt viruses^1^. Heavy BPH infestation causes serious damage to rice crop as shown by symptoms of complete drying and mortality known as “hopper burn”. In recent years, infestations of BPH have intensified in many countries as BPH developed the ability to attack resistant plants and gained resistance to widely used pesticides. Previous studies showed that host-plant resistance is an effective, environment-friendly approach to reducing BPH damage and increasing yield potential. To date, 30 BPH resistance loci have been reported from cultivated rice germplasm and as well from five wild *Oryza* species sources^2^,^3^. Among these, the *Bph3, Bph14, BPH26* and *BPH29* genes have been identified by map-based gene cloning. The *Bph3* locus was revealed to be a cluster of three genes encoding plasma membrane-localized lectin receptor kinases (OsLecRK1-OsLecRK3)^4^. The *Bph14* gene encodes a protein containing a coiled-coil nucleotide-binding site (CC-NBS) and a leucine-rich repeat (LRR) motif, and mediates a resistance mechanism similar to the defense mechanism against pathogens through the activation of the salicylic acid (SA)-dependent pathway^5^. The *BPH26* gene also encodes a CC-NBS-LRR protein, and mediated sucking inhibition in the phloem sieve element^6^. The *BPH29* encoding B3 DNA-binding domain confers BPH resistance through activation of SA pathway^7^.

The mechanism of host resistance to a broad range of BPH populations is still elusive. Innate immune response plays a critical role in the survival of plants against pathogens or insects. Plants have developed two strategies of immunity against attack of pathogens: pathogen-associated molecular patterns (PAMPs)-triggered immunity (PTI) and effector-triggered immunity (ETI)^8^. On the external face of the host cell, conserved microbial elicitors called PAMPs are recognized by receptor proteins, which trigger PTI. Pathogens evolve to suppress PTI by secreting virulence molecules called effectors into the host cell. The recognition of effector proteins by resistance (R) proteins induces ETI. Receptor kinases and a set of NBS-LRR proteins are involved in recognizing PAMPs or effectors and turning on the host-resistance pathways. In rice, most of the cloned *R* genes encode CC-NBS-LRR type proteins or receptor kinases^4^,^5^,^6^,^8^,^9^,^10^,^11^. Several effector genes in rice pathogens of blast and bacterial blight have been revealed^12^.

Plants also have developed elaborated protection systems against herbivore attack. The herbivore-associated molecular patterns (HAMPs) or the herbivore associated elicitors (HAEs) are recognized by plant cells, which triggers signal transduction pathways that connect herbivore-specific elicitors to the expression of suitable defense genes^13^. Various elicitors in the insects’ oral secretions have been discovered and have been well reviewed by Wu and Baldwin^14^. Recently, candidate effectors which appear to elicit plant defenses or promote plant-insect interactions have been reported^15^,^16^. It was proposed that HAMP-triggered immunity (HTI) and ETI are also applicable to plant-insect interactions^15^.

Phloem-feeding insects (PFIs) such as planthoppers, aphids, and whiteflies have specialized mouthparts and stylets that navigate through the apoplastic space of different cell layers, allowing them to reach phloem cells, puncture and ingest the sap. PFIs initially secrete sheath saliva, which is hypothesized to form a protective layer around stylets, and watery saliva during probing and feeding, which is thought to be involved in modulating the host-cell process^16^. Several genes conferring resistance to PFIs have been identified (tomato *Mi-1* encoding CC-NBS-LRR protein^17^, melon *Vat* encoding CC-NBS-LRR protein^18^ as well as the above four rice BPH resistance genes^4^,^5^,^6^,^7^).

It is imperative to identify more BPH-resistance genes to elucidate their interactions for understanding the mechanism of resistance toward the development of durable broad-spectrum BPH-resistant varieties. In rice breeding programs, *BPH18* was utilized to breed a BPH resistant variety in japonica background through marker-assisted selection. The variety, Anmi, harboring *BPH18* showed BPH resistance at the seedling as well as at adult stages in Korea^19^. In this study, we report that the *BPH18* gene, a unique resistance gene derived from a distantly related wild *Oryza* species (*O. australiensis*)^1^, encodes a novel type of CC-NBS-NBS-LRR protein and confers BPH resistance.

## Results

### Map-based cloning and complementation test revealed that *BPH18* encodes a CC-NBS-NBS-LRR protein

**Figure 1:**
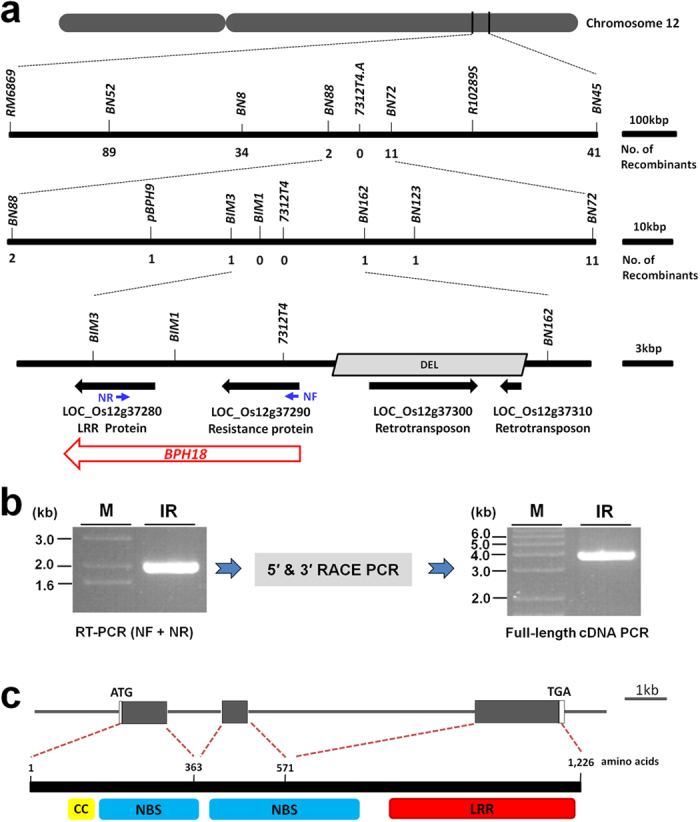
Map-based cloning of the *BPH18* gene. (**a**) Fine mapping of *BPH18*. Numbers under the linkage map indicate the number of recombinants detected between the markers at the *BPH18* locus. Gene models annotated in the Rice Genome Annotation Project database (http://rice.plantbiology.msu.edu/, RGAP 7 version) were shown as black filled arrows and the actual *BPH18* gene was shown with a red arrow. The grey parallelogram indicates the genomic region which is absent in the resistance donor line, IR65482, compared with Junam. NF and NR represent primers for testing a combined gene model. (**b**) Identification of the *BPH18* full-length cDNA from the resistant line. RT-PCR was conducted with NF and NR primers. And RACE PCRs were done to determine 5′ and 3′ ends of cDNA. Finally 3,934 bp of the *BPH18* full-length cDNA was obtained by PCR with NFCF and NFCR primers. M: DNA size marker, IR: IR65482. (**c**) The *BPH18* genomic structure and the BPH18 protein structures of the resistance donor line. The filled-gray boxes indicate the protein coding region and the blank boxes indicate 5′ and 3′ un-translated regions (UTRs). The deduced BPH18 protein consists of 1,226 amino acids and has a CC domain, two NBS domains, and an LRR domain.

In our previous study, *BPH18* was identified in an introgression line IR65482-7-216-1-2 (designated as IR65482 hereinafter), inheriting the gene from the wild species *O. australiensis*^1^. *BPH18* was mapped in an 843-kb interval between the markers *R10289S* and *RM6869* and completely co-segregated with marker *7312.T4A* on the long arm of chromosome 12^1^. For the fine-mapping of *BPH18*, we planted 3,100 BC_4_F_2_ plants derived from the Junam/IR65482 cross and genotyped all the plants with two markers, *BN45* and *BN52, w*hich were developed at about 450-kb distance forward and backward of the marker *7312.T4A*. We identified 149 plants that had recombinant genotype in the *BN45* - *BN52* interval. The BC_4_F_3_ progenies from the selected 149 BC_3_F_2_ plants were genotyped again with *BN45* and *BN52* to select plants that have homozygous recombinant genotype in this interval. Thus, 130 BC_4_F_3_ plants were selected and their seeds were harvested. The BC_4_F_4_ plants from these selected homozygous recombinant 130 BC_4_F_3_ plants were subjected to BPH bioassay, and the selected BC_4_F_3_ lines were genotyped with additional markers in the target region. These analyses revealed that the *BPH18* was delimited to a 27-kb region based on the Nipponbare genome sequence flanked by the markers *BIM3* and *BN162* (Fig. 1a). In this region, four genes were annotated in the Rice Genome Annotation Project database (http://rice.plantbiology.msu.edu/): *LOC_Os12g37280* annotated as an LRR protein gene, *LOC_Os12g37290* annotated as a resistance protein gene containing the NBS domain, and *LOC_Os12g37300* and *LOC_Os12g37310* annotated as retrotransposon genes. Sequencing the region from IR65482 and Junam revealed that a 14-kb region including the two retrotransposon genes were absent in IR65482. The remaining region contained the *LOC_Os12g37280* and the *LOC_Os12g37290* in IR65482 (Fig. 1a).

**Figure 2:**
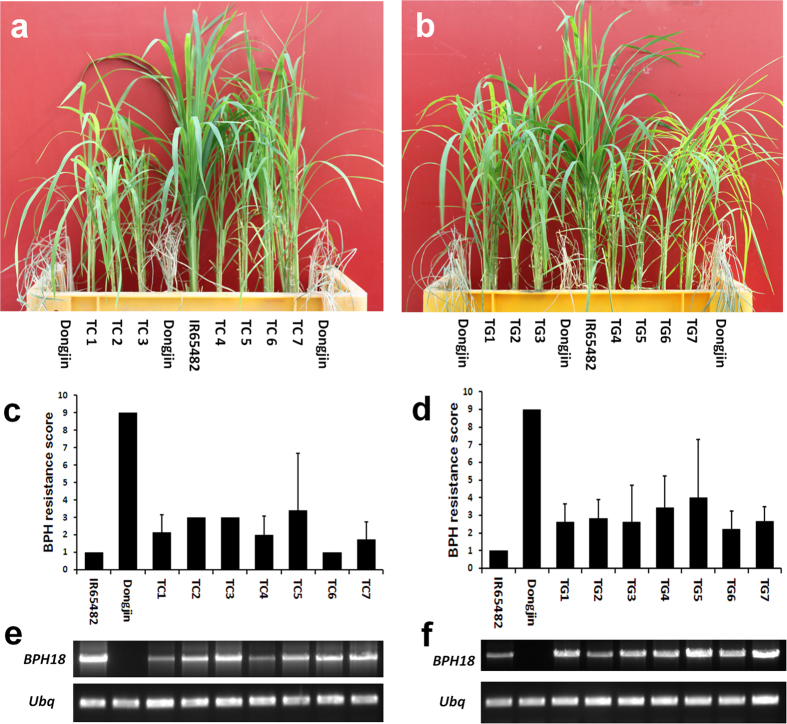
Complementation test of the *BPH18*. (**a**,**b**) BPH bioassay of the *BPH18* transgenic lines (T_1_ generation). IR65482, resistant parental line; Dongjin, susceptible background variety; TC1-7, transgenic lines harboring the fusion gene of *BPH18* promoter*::BPH18* ORF*::BPH18* terminator; TG1-7, transgenic lines harboring the full *BPH18* genomic region (14 kb), including its promoter and terminator. (**c**,**d**) BPH resistance scores of the *BPH18*-transgenic lines at the seedling stage. Lower scores indicate a higher resistance to the insect. The BPH resistance score of the rice seedlings was evaluated according to a method described by Huang *et al*.^53^. Data are means ± standard deviation. (**e**,**f**) RT-PCR analysis of *BPH18* in the transgenic T_0_ lines. The *BPH18* primer pair flanked the whole ORF (3,681bp). *Ubiquitin (Ubq*) gene was used as an internal control.

Between the two genes, we set the *LOC_Os12g37290* encoding the NBS domain protein as the candidate gene for *BPH18* and did complementation experiment. The 6.4-kb genomic region of *LOC_Os12g37290*, including promoter and terminator from the resistant line (Supplementary Fig. S1), was transferred into the susceptible japonica variety, Ilmi. The deduced protein of *LOC_Os12g37290* had the NBS domain (Supplementary Fig. S1), but did not carry the LRR domain that is present in most NBS-LRR R proteins at the C-terminal regions^20^. However, the transgenic plants did not show enhanced resistance (Supplementary Fig. S2). Therefore, we speculated that *LOC_Os12g37290* encoding the NBS domain and *LOC_Os12g37280* encoding the LRR domain will form one gene to encode the usual NBS-LRR protein. To test this, reverse transcription PCR (RT-PCR) was conducted with a forward primer (NF) in *LOC_Os12g37290* and a reverse primer (NR) in *LOC_Os12g37280*. This resulted in a clear PCR product (Fig. 1b), suggesting that two ORFs predicted is due to a false annotation. The full-length cDNA of the combined gene model was identified by 5′ RACE and 3′ RACE PCR (Fig. 1b). The gene consists of three exons encoding a protein of 1,226 amino acids with a CC motif, two NBS domains, and a LRR motif (Fig. 1c, Supplementary Fig. S3). We further examined the gene encoding CC-NBS-NBS-LRR, its ORF from IR65482 was isolated and placed between its own promoter and terminator regions (Supplementary Fig. S4a) and introduced into a susceptible cultivar Dongjin. Transgenic plants that expressed the introduced genes showed enhanced BPH resistance compared to the parental line (Fig. 2a,c,e), indicating that the gene encoding CC-NBS-NBS-LRR protein confers BPH resistance. This was confirmed by the introduction of the 14-kb genomic fragment covering the entire gene from IR65482 (Supplementary Fig. S4b). The transgenic lines also showed enhanced resistance to BPH (Fig. 2b,d,f). Additional functional evidence was provided by generating *BPH18*-RNAi transgenic plants in the resistant introgression line. Of the six RNAi lines, five lines showing a suppressed expression of *BPH18* displayed significantly reduced BPH resistance (Supplementary Fig. S5). These data conclude that the gene encoding CC-NBS-NBS-LRR protein is *BPH18* gene and is responsible for BPH resistance.

The sequence comparison of *BPH18* between the resistant donor line and the susceptible variety (Junam) revealed that the susceptible allele lacked the last part of the second NBS domain and the whole LRR domain due to premature stop codon in the beginning of the third exon (Supplementary Fig. S6). The absence of the conserved domain of BPH18 may make Junam susceptible to BPH.

### BPH18 is close to the BPH26 and its first NBS domain is partial

**Figure 3:**
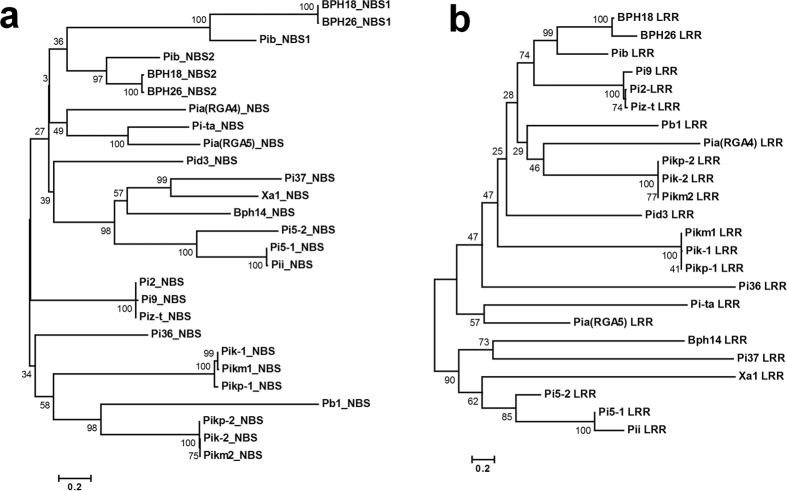
Phylogenetic relationship of BPH18 and other identified rice NBS-LRR R proteins. (**a**) Phylogenetic tree based on NBS domain sequences. The sum of branch length was 14.612. (**b**) Phylogenetic tree based on LRR domain sequences. The sum of branch length was 23.078. Phylogenetic relationship was reconstructed using neighbor-joining distance method. Node supports are given in percentage of 1000 bootstrap replicates. Branch lengths are proportional to phylogenetic distances estimated from JTT amino acid substitution model.

Phylogenetic analysis of BPH18 with previously identified rice NBS-LRR R proteins based on the NBS domain and LRR domain sequences showed that BPH18 is closest to BPH26 cloned from chromosome 12, and they exhibit highest similarity to the Pib protein conferring rice blast resistance located on chromosome 2^21^ (Fig. 3a,b). These three proteins have two NBS domains unlike other typical NBS-LRR proteins. Bph14, which was the first identified BPH-resistance protein in rice^5^, was much farther related with BPH18 and BPH26 (Fig. 3a,b). Among these R proteins, LRR domain sequences are much more divergent than NBS domain sequences (The sum of branch length of the phylogenetic tree based on NBS domain sequences was 14.612 while that based on LRR domain sequences was 23.078).

A typical NBS domain of R proteins contains three sub-domains; a core NB (nucleotide binding), and two ARC sub-domains^22^,^23^. On the other hand, the second NBS regions of BPH18, BPH26 and Pib have all three conserved sub-domains, with the first NBS regions carrying only the NB sub-domain and lacking a portion of ARC1 and entire ARC2 (Supplementary Fig. S7). The first NBS domains of BPH18 and BPH26 have P-loop, RNBS-A, kinase-2, RNBS-B, and RNBS-C motifs but lacked GLPL, RNBS-D, and MHD motifs, which are in the ARC sub-domains^24^. The P-loop motif of the first NBS domain is much different from the consensus sequences. While the consensus sequence is GMGGIGKTT, that of BPH18 and BPH26 is GTSGDIREMS. Considering that the P-loop is the most-conserved motif in the NBS domains and that the lysine (K) and threonine (T) residues within the domain bind to ATP and a Mg^2+^ ion^22^, the first NBS domain is likely nonfunctional or has evolved to a diversified function.

### The *BPH18* expression pattern was consistent with BPH insect feeding site

**Figure 4:**
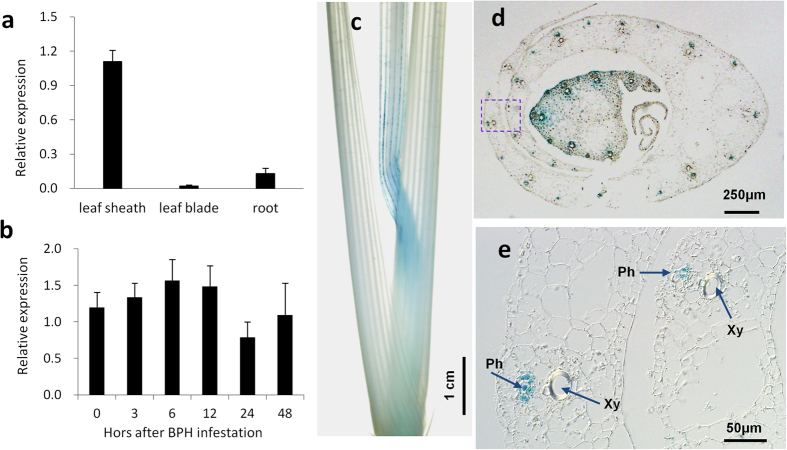
Expression analyses of *BPH18* using qRT-PCR and GUS reporter system. (**a**) Expression of *BPH18* in the leaf sheath, leaf blade and root. The mean was calculated based on the average of four biological repeats. The expression level in the samples was quantified relative to the first replicate of time 0. (**b**) Time-course expression of *BPH18* in the leaf sheath before and after BPH infestation. The 0 means the time point just before BPH infestation. The mean was calculated based on the average of five biological repeats. The expression level in the samples was quantified relative to the first replicate of root. (**c–e**) GUS expression driven by the *BPH18* promoter. Relatively strong GUS activity was detected in the leaf sheath (**c**). The leaf sheath was cross-sectioned (**d**). The rectangle region in Fig. 4d was magnified (**e**). Ph, phloem; Xy, xylem.

We investigated the expression pattern of *BPH18* using quantitative real-time PCR (qRT-PCR) and found that its transcript levels mainly found in leaf sheathes and weakly detected in leaf blades and roots (Fig. 4a). *BPH18* was expressed before and after BPH infestation, indicating that it is expressed constitutively (Fig. 4b). To study a detailed expression pattern of the gene, we produced the *BPH18* promoter-*GUS* transgenic plants. Histochemical analysis revealed that strong GUS activities were detected in the vascular bundles of the leaf sheath (Fig. 4c), where a BPH’s stylet targets.

### BPH18 localized to endo-membranes

**Figure 5:**
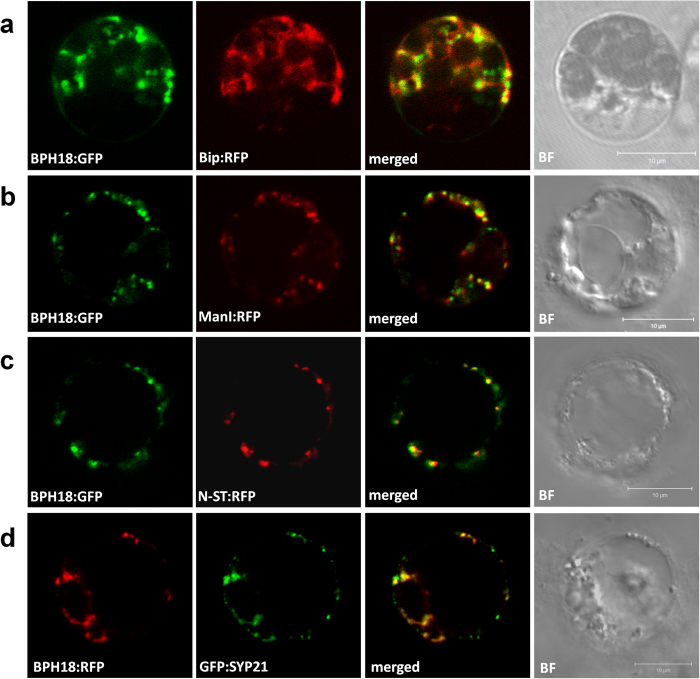
Subcellular localization of BPH18 protein. BPH18 proteins co-expressed with endoplasmic reticulum maker (Bip:RFP) (**a**), *cis*-Golgi marker (ManI:RFP) (**b**), *trans*-Golgi network marker (N-ST:RFP) (**c**), and prevacuolar compartment marker (GFP:SYP21) (**d**). Protoplasts were prepared from rice seedling shoots (**a**) and rice Oc cell line (**b–d**). Fluorescence signals were detected from the protoplasts under confocal microscopy. BF, bright field; Scale bar = 10 μm.

The subcellular localization of BPH18 was investigated through its transient expression fused with Green fluorescent protein (GFP) or Red fluorescent protein (RFP) in rice protoplasts. BPH18:GFP and BPH18:RFP showed both reticular and punctate patterns in contrast to free GFP and RFP patterns which were localized to cytosol (Supplementary Fig. S8). To investigate the localization of BPH18 in detail, we co-expressed BPH18:GFP with an endoplasmic reticulum (ER) marker (Bip:RFP)^25^, *cis*-Golgi marker (ManI:RFP)^26^, and *trans*-Golgi network (TGN) marker (N-ST:RFP)^27^. We also co-expressed BPH18:RFP and prevacuolar compartments (PVC) marker (GFP:SYP21)^28^. The BPH18 fluorescence signals were co-localized partly with the ER, Golgi, TGN, and PVC markers (Fig. 5a–d), suggesting that the BPH18 protein is widely distributed to various endo-membranes, including ER, Golgi, TGN, and PVC.

### *BPH18* involved in both antibiosis and antixenosis resistance mechanism

**Figure 6:**
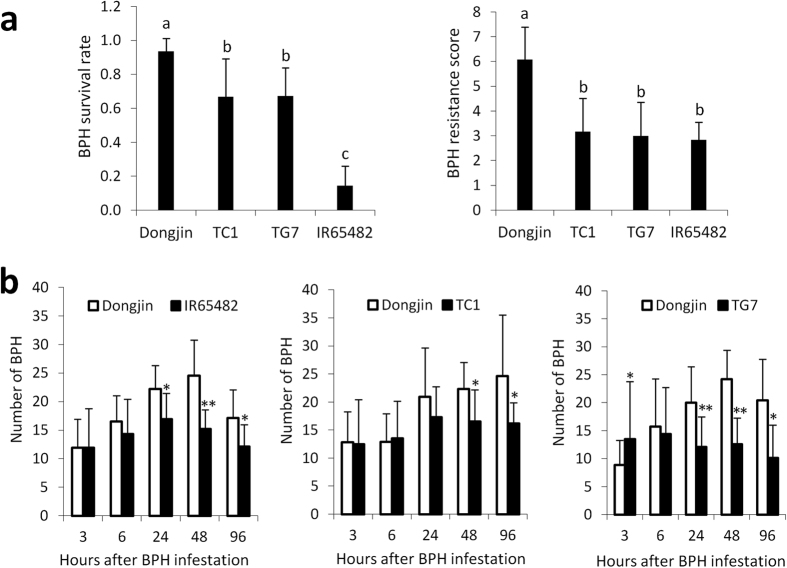
Antibiosis and antixenosis effect of *BPH18*. (**a**) Antibiosis effect of *BPH18* was measured by BPH survival rate. Four-week-old seedlings were transferred in a glass tube with 7–11 nymphs of BPH per plant, and BPH survival rates were measured at seven days after BPH infestation when the susceptible variety began to die. The average values were obtained from twelve replications, and the error bar shows standard deviation. Statistical tests of difference among survival rates were done using Tukey’s honestly significant difference test. The letters on the bars represent groups, in which observations were not significantly different. The BPH resistance score of tested lines are shown on the graph at the right. The BPH resistance score of the rice seedlings was evaluated according to a method described by Huang *et al*.^53^. (**b**) Antixenosis effect of *BPH18* measured by the host choice test. The number of BPH nymphs that settled on rice seedlings was shown at a time course of 3, 6, 24, 48, and 96 h after BPH were released into the pot covered with a light-transmitting mesh. Asterisks on bars represent significant difference between two lines by *t*-test (★α = 0.05, ★★α = 0.01).

Of the three different mechanisms of resistance to BPH (antibiosis, antixenosis and tolerance)^7^, rice plants employ two major resistance strategies against herbivores^5^: antibiosis, which reduces insect feeding, growth rate, or survival, and antixenosis, which affects insect settling, colonization, or oviposition. To investigate the mechanism of resistance, we evaluated the *BPH18* complementation transgenic lines, TC1 and TG7, along with the susceptible variety Dongjin and resistant donor line IR65482. The cultivar Dongjin showed a high BPH survival rate while the transgenic lines showed a significantly lower BPH survival rate (Fig. 6a). The antixenosis effect of *BPH18* was assessed by the host choice test, which showed a significantly less number of BPH settling on rice seedlings in the transgenic plants compared to the susceptible Dongjin at 24–96 h after BPH infestation (Fig. 6b). These results suggest that *BPH18* confers resistance via both antibiosis and antixenosis effects.

### *BPH18* and *BPH26* are in the same genomic location but they are functionally different alleles

**Figure 7:**
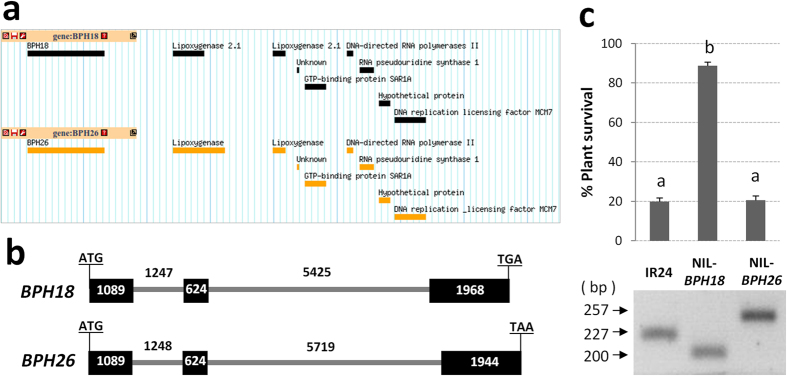
Comparison of genomic locus, gene structure, and BPH reaction between *BPH18* and *BPH26*. (**a**) Genomic structures near the *BPH18* (top) and *BPH26* (bottom) loci. The surrounding genes were annotated based on the whole genome sequence data of NIL-*BPH18* and NIL-*BPH26*. Scale = 1 kb. (**b**) Gene structures of *BPH18* and *BPH26* from translation start codon to stop codon. Filled black boxes indicate protein coding region. The size (bp) of exon and intron were shown with number. (**c**) The BPH bioassay result of susceptible recurrent variety (IR24), NIL-*BPH18*, and NIL-*BPH26*. The percent of plant survival was observed after BPH infestation. The average was calculated from two seasons with replications. Error bar means standard deviation. Statistical difference shown as *a* or *b* was obtained through least significant difference test (α = 0.01). The sequence difference in the second intron among NIL-*BPH18*, NIL-*BPH26*, and IR24 was confirmed by PCR with the BPH18-ind2 primer set.

In a recent study, another BPH resistance gene, *BPH26,* which is located at the *BPH18* locus on chromosome 12 was cloned^6^. To reveal the relationship between these two genes, we developed NILs of *BPH18* and *BPH26* in the BPH-susceptible indica variety IR24. The whole genome sequencing of NILs (*BPH18* and *BPH26)* and IR24 showed the integration of the donor segments including *BPH18* or *BPH26* gene into IR24 background (Supplementary Fig. S9). We analyzed the genomic structure of the surrounding regions of the *BPH18* and *BPH26* locus, respectively. The arrangement of the surrounding genes were quite similar between *BPH18* and *BPH26* loci (Fig. 7a), resulting that both genes located at the same locus. However, *BPH18* and *BPH26* showed remarkable differences at the genomic sequence level (Supplementary Fig. S10), despite both having three exons and two introns each. The length of the second intron and the third exon showed a 294-bp and 24-bp differences, respectively (Fig. 7b). The sequence difference was also confirmed by PCR in two NILs (Fig. 7c). In the protein coding sequence, *BPH18* and *BPH26* have 195 single nucleotide polymorphisms (SNPs) and four gaps (Supplementary Fig. S10). At the amino acid sequence level, we found 105 amino acids difference and five gaps, with 88 of the amino acids difference and all five gaps detected in the LRR domain (Supplementary Fig. S11). To compare the function of alleles, we performed the BPH bioassay in NIL-*BPH18* and NIL-*BPH26* plants with the BPH insects collected in Nueva Ecija Province, Philippines. The NIL-*BPH26* was susceptible to this BPH biotype as IR24 but NIL-*BPH18* showed clear resistance (Fig. 7c). These results indicate that *BPH18* and *BPH26* are functionally different alleles even though they are located at the same locus.

Plant defense responses to insects involve global changes in gene expression mediated by plant hormone SA and jasmonic acid (JA)/ethylene signaling pathways^29^. To investigate the pathway involved in the BPH resistance of *BPH18*, we observed the expression changes of the defense-related genes in IR24 and the two NILs after BPH infestation using qRT-PCR (Supplementary Fig. S12). Overall gene expression patterns of the plant defense-related genes were similar between the susceptible lines, IR24 and NIL-*BPH26* and it differed with NIL-*BPH18*. In the susceptible lines, JA synthesis-related genes (*LOX* and *AOS2*), in SA synthesis-related gene (*EDS1*), ethylene receptor gene (*EIN2*), and a pathogen-related gene (*PR1b*) were strongly increased, especially at 72 h after BPH infestation. In contrast, in NIL-*BPH18*, none of the defense-related genes tested in this study was strongly activated by BPH insect, suggesting that unknown pathway may be involved in BPH resistance in NIL-*BPH18*. However, the gene expression patterns of the defense-related genes were significantly different between NIL-*BPH18* and NIL-*BPH26*.

## Discussion

As a PFI, the BPH probes intercellular plant tissues to establish feeding sites in the phloem sieve elements^30^. Remarkable similarities between plant responses to phloem feeders and pathogens have been found^31^. To date, six PFI-resistance genes (*Mi-1, Vat, Bph3, Bph14, BPH26,* and *BPH29*) have been isolated in plants. Four of these encode CC-NBS-LRR proteins which are typical among plant R proteins against pathogens. BPH18 and BPH26 proteins have a CC-NBS-NBS-LRR domain structure, which is basically similar to the typical CC-NBS-LRR R proteins. This commonality among the PFI-resistance proteins and R proteins against plant pathogens might indicate the similar molecular mechanism in resistance. As intracellular receptors, NBS-LRR R proteins sense pathogen effectors directly or host protein modifications induced by pathogen molecules while pathogens secrete into the plant cell to suppress immune response and trigger potent innate immune responses^20^,^32^,^33^,^34^. Additionally NBS-LRR proteins as helpers of defense signaling transduce signals downstream of some pathogen-activated NBS-LRR proteins^35^. PFIs puncture the phloem cell and secret watery saliva that contains complex mixtures of lipoproteins, phospholipids, and carbohydrates, as well as numerous enzymes with proteolytic, hydrolytic, oxidative, or cell wall-degrading activities^30^. These factors probably aid in stylet penetration and could detoxify defensive compounds in the host plant^30^. The PFI oral secretions are a potential source of effectors or avirulence (avr) factors. Elucidation of the molecular mechanism of BPH effectors corresponding with *Bph14, BPH18, BPH26* and *BPH29* is needed to get basic knowledge on resistance to BPH.

The *BPH18/BPH26* and *Pib* encode R proteins of the unique domain structure of CC-NBS-NBS-LRR having two NBS domains with unconserved P-loops, respectively. Of the 480 NBS-LRR genes identified in the japonica rice genome^36^, only four genes encode proteins having two NBS domains in which NBS domains are partially duplicated. The first NBS domains of BPH18/BPH26 and Pib are partial, lacking most of the ARC sub-domains, and their P-loop motifs were much different from the consensus sequence. When NBS-LRR proteins function as a helper which regulates signal transduction following pathogen effector recognition by other NBS-LRR proteins, the P-loop is not essential for these functions. For example, rice Pb1 conferring durable resistance to neck blast disease^37^, its orthologue in *Arabidopsis* ADR1-L2 involved in bacterial pathogen resistance^35^ might be regarded as helper NBS-LRR proteins for defense signaling. They encode a CC-NBS-LRR protein having a degenerate P-loop in the NBS domain. These types of NBS-LRR proteins need to be studied to check whether they function as sensor, helper, or both.

Plants deploy intracellular immune receptors such as NBS-LRR proteins to perceive pathogen invasion. Through the subcellular localization experiments, it is revealed that BPH18 is localized widely to endo-membranes, including ER, Golgi, TGN, and PVC. Plant NBS-LRR R proteins reside in diverse subcellular locations, and each R protein will be in the place of its effector or effector target^23^. Membrane trafficking is emerging as a central theme in plant innate immunity and has been implicated in immune receptor activation, defense signaling, and targeting of cellular cargo to pathogen invasion sites^38^. Critical components of host-membrane trafficking are prime targets of pathogen effectors^39^. The *Xanthomonas campestris* pv. *vesicatoria* type III effector protein XopJ is localized in the Golgi body and plasma membrane, suppressing protein secretion and callose deposition, which leads to the weakening of cell wall-associated defense responses^40^. A *Pseudomonas syringae* virulence protein, HopM1, mediates the destruction of an immunity-associated protein, AtMIN7, which is involved in vesicle trafficking pathway and cell wall-associated defense; both HopM1 and AtMIN7 are localized to the *trans*-Golgi network/early endosome^41^,^42^. The ARFA1b/c, an ARF GTPase localized in the multi-vesicular body/PVC, is required for callose deposition for pre-invasive penetration resistance against powdery mildew in barley^43^. The *Arabidopsis* TIR-NB-LRR R protein, RPP1A, which confers resistance to the oomycete *Hyalopernospora parasitica*, resides in the ER/Golgi apparatus^44^. Based on these examples, we hypothesize that the *BPH18* protein is possibly involved in recognizing the invasion of BPH and in detecting some effector proteins of BPH, which targets endo-membranes and interferes with the vesicle trafficking pathway and cell wall-associated defense, including callose deposition.

Even though the transgenic lines harboring *BPH18* showed significant antibiosis effect, the effect was weaker than in the original resistant donor line. However, *BPH18* showed their antixenosis effect in transgenic lines, which was similar with the resistant donor (Fig. 6a,b). *Bph6* from the indica rice variety Swarnalata also had antibiosis and antixenosis effects^45^. Interestingly, *Bph6* conferred a higher resistance when it was introgressed into an indica-susceptible genetic background than when it was introgressed into a japonica-susceptible genetic background. This might explain why *BPH18* transferred into a japonica variety showed a weaker antibiosis effect than the original indica resistance donor line. One another possible explanation is that at least two more minor Quantitative Trait Loci (QTLs) were found on the short arm of chromosome 5 and on the end of chromosome 12 in the previous *BPH18* mapping study^1^. Incorporating these QTLs would further enhance BPH resistance of *BPH18* harboring rice lines.

*BPH18* and *BPH26* are on the same locus in the long arm of chromosome 12. Similarly the *Pi2, Piz-t*, and *Pi9* genes are in the same genomic region of rice chromosome 6 including a cluster of nine NBS-LRR genes^10^,^46^. *Pi2* and *Piz-t* are two different resistant alleles by eight amino-acid differences for the fourth NBS-LRR gene in this cluster, and *Pi9* corresponds to the second NBS-LRR gene. Whole genome sequencing of the NIL-*BPH18* and NIL-*BPH26* revealed that *BPH18* and *BPH26* located at the same locus (Fig. 7a). This might imply that *BPH18* and *BPH26* are the same genes with different alleles. Unlike the *Pi2* and *Piz-t* alleles, many DNA and amino acid sequence differences were found between the *BPH18* and *BPH26* genes. This may be derived from evolutionary divergence between the donor sources of these two genes. While *BPH26* came from *O. sativa* indica variety ADR52, *BPH18* was originated from *O. australiensis*.

The amino acid sequences of LRR domains of BPH18 and BPH26 are very much divergent while their NBS domains are well conserved (Supplementary Fig. S11), which suggests that LRR domains determine resistance specificities in the case of BPH18 and BPH26. The LRR domains of NBS-LRR proteins recognize pathogen effectors directly and determine resistance specificities. The LRR domain of Pi-ta binds directly with its cognate fungal effector Avr-Pita, and a single amino acid difference in the LRR domain of Pi-ta distinguishes resistant and susceptible alleles^9^,^47^. The eight amino-acid differences within the LRR domains between Pi2 and Piz-t determine resistance specificity^46^. In comparison of the *L6* and *L11* alleles of flax, polymorphisms in LRR domains account for the specific recognition of AvrL567 by L6 and AvrL11 by L11^48^,^49^. Also, it was revealed that six amino acid changes confined to LRR domains determine the difference between P and P2 rust resistance specificities in flax^50^. The *in planta* association of *Arabidopsis* RPP1 resistance protein and its cognate oomycete effector ATR1 was mediated by the LRR domain of RPP1^51^. In addition to discovering effectors of BPH, molecular mechanism of their recognition by BPH resistance proteins should be elucidated, and their LRR domains might be strong candidates for effector recognition domains.

The NIL-*BPH18* and NIL-*BPH26* in the same susceptible background, IR24, showed different resistance reactions to the BPH strain collected in Nueva Ecija Province, Philippines. While, the NIL-*BPH26* showed susceptibility like IR24, NIL-*BPH18* showed higher resistance to BPH. Similar result was observed in the previous report. Neither *BPH26* nor *BPH25* in susceptible Taichung65 background showed resistance to the BPH strain, Japan-KG-06 which is *BPH2*-virulent biotype. When they coexist, the line showed resistance to that biotype^6^. However, *BPH18* without *BPH25* showed resistance to the Nueva Ecija BPH population, supporting that this BPH insect belongs to *BPH2*-virulent biotype, and *BPH18* and *BPH26* are functionally different alleles. Different resistance reactions to the same BPH population suggests that BPH18 and BPH26 recognizes the different effectors or the modifications of different plant proteins caused by effectors through the variable LRR domain probably. Gene expression patterns of the plant defense-related genes were quite different between the NIL-*BPH26* and NIL-*BPH18*. This result indicates that the two BPH resistance genes utilize different resistance pathways after BPH attacks. It was observed that in *Bph14* and *BPH29* lines, JA synthesis-related genes are not upregulated but SA synthesis-related genes were strongly expressed by BPH infestation^5^,^7^. In NIL-*BPH26*, both SA and JA synthesis-related genes were strongly induced by BPH, suggesting that *BPH26* may activate JA and SA-dependent resistance pathway. In NIL-*BPH18*, there was no significant transcription activation of the previously identified defense-related genes. Similarly, RNA sequencing experiment revealed that the transcript level of SA dependent pathway genes are not different between NIL-*BPH15* and its susceptible recipient line^52^. But the expression level of many regulatory genes including hormone signaling genes, receptor kinases, and transcription factors were changed by BPH infestation. Like *BPH15*, unidentified resistance pathway may lead the BPH resistance in NIL-*BPH18*.

Transfer of the *BPH18* gene conferring resistance could control planthopper infestation in rice. The *BPH18* gene derived from wild rice should be utilized in breeding programs as a new source of resistance to increase rice production.

## Methods

### BPH insect materials

The BPH insects were collected from rice fields in South Korea in 2003. A pure BPH population was developed from a single colony of BPH and was grown on the susceptible japonica variety Taebaekbyeo in a glass house^1^. This Korean BPH biotype was used for fine-mapping and the experiments with transgenic plants in Suwon, South Korea. At IRRI, Philippines, we used the BPH population collected in 2011 from Nueva Ecija Province which is the major rice cultivation area in the Philippines. A pair of BPH insects was collected and cultured in the glass house on the susceptible variety, T(N)1. After culturing and maintaining several generations, this BPH population was used for evaluation of NIL-*BPH18* and NIL-*BPH26*.

### Fine-mapping of *BPH18*

The 3,100 BC_4_F_2_ plants derived from the cross between the susceptible cultivar Junam and IR65482-7-216-1-2 as a resistant donor were used as the fine-mapping population. We developed two cleaved amplified polymorphic sequences (CAPS) markers, *BN45* and *BN52,* flanking the *BPH18* region of about 1.1 Mb. We genotyped all the plants with these two markers and selected the plants that had a recombinant genotype in this interval. A total of 149 plants were selected. The BC_4_F_3_ lines from these selected plants were planted, each line comprising 13 plants. All the plants of this BC_4_F_3_ population were genotyped again with the two flanking markers from which 130 plants that had homozygous recombinant genotype were selected. Seeds from the selected plants were harvested and BC_4_F_4_ progenies were sown for BPH bioassay. The bioassay was done with the Korean BPH biotype using the modified bulk seedling test (MBST) method^1^. Seedlings at the three-leaf stage were infested with second- or third-instar nymphs at a density of 10–12 nymphs per seedling. When all the seedlings of the susceptible control died, the tested plants were evaluated as resistant or susceptible depending on survival or death of seedlings. Eight CAPS and InDel markers (Supplementary Table S1) in the *BN52-BN45* region were used for genotyping the selected homozygous recombinant BC_4_F_3_ plants. The 27-kb genomic region, revealed to harbor *BPH18* by fine-mapping in the *BPH18* donor line, was sequenced.

### Identification of the *BPH18* full-length cDNA

To test whether the *LOC_Os12g37280* and *LOC_Os12g37290* genes make one gene encoding a CC-NBS-NBS-LRR protein, the NF and the NR primers were used for RT-PCR with cDNA synthesized from the RNA of the *BPH18* donor line. To identify the full-length cDNA of this combined gene model, 5′ RACE and 3′ RACE PCR was conducted using the CapFishing^TM^ Full-length cDNA Premix Kit (Seegene, Korea). The full-length cDNA of *BPH18* was confirmed by PCR with NFCF and NFCR primers. All primers for molecular analysis of *BPH18* gene are shown in Supplementary Table S2.

### Complementation test and RNAi experiment

Firstly, a 6.4-kb genomic DNA fragment of the *LOC_Os12g37290* gene in the *BPH18* donor was amplified with RPL2F and RPL2R primers and finally it was inserted into the pCAMBIA1300 binary vector. The BPH-susceptible japonica variety, Ilmi, was transformed with this construct using the *Agrobacterium*-mediated method. The T_1_ plants were subjected to BPH bioassay. Based on the new gene model, combining *LOC_Os12g37290* and *LOC_Os12g37280*, the promoter, ORF, and terminator part were amplified with BPH18-pro, BPH18-ORF, and BPH18-ter primer pairs, respectively, then inserted into pPZP vector consecutively. Alternatively the 8-kb genomic region, including the *LOC_Os12g37280* gene in the *BPH18* donor, was amplified with a pair of LRR-8.0 primers. The purified PCR product was inserted into the already constructed pCAMBIA1300 vector, harboring the *LOC_Os12g37290* gene with In-Fusion HD Cloning Kit (Clontech, USA). Thus, the constructed vector included the whole 14-kb genomic region of the *LOC_Os12g37290* gene and *LOC_Os12g37280* gene of the *BPH18* donor. The BPH-susceptible japonica variety, Dongjin, was transformed with this construct and the T_1_ plants having the transgene were selected by PCR or *bar*-strip test using the AgraStrip LL Rice Strip Test Kit (Romer Labs, USA). Then, the plants were subjected to BPH bioassay with the South Korea BPH strain. To generate the RNAi construct for the *BPH18* gene, we amplified a 435-bp fragment of *BPH18* donor cDNA using primers BPH18i-F and BPH18i-R. Finally the fragments were cloned into the destination vector, pANDAβ. The BPH-resistant NILs in Junam background, NIL-*BPH18*, was transformed with this construct and the T_2_ plants having the RNAi construct were subjected to BPH bioassay. The BPH resistance score of the rice seedlings was evaluated according to the method described by Huang *et al*.^53^.

### Domain structure and phylogenetic analysis

The domain structure of BPH18 was analyzed using the CD SEARCH program of the NCBI website (http://www.ncbi.nlm.nih.gov/). The CC structure was predicted by Paircoil2^54^ program (http://groups.csail.mit.edu/cb/paircoil2/). The amino acid sequences of the identified rice NBS-LRR R proteins and human APAF-1 protein were downloaded, and their NBS and LRR domain sequences were used for sequence alignment and phylogenetic analysis. The protein alignment was generated with ClustalW^55^. We used MEGA 6.0^56^ to reconstruct neighbor-joining trees. For the tree analysis, we performed 1000 bootstrap replicates to assess the support for the nodes.

### Gene expression analysis of *BPH18*

Five-leaf-stage plants of the resistance donor line (IR65482-7-216-1-2) were infested with the South Korea BPH strain and sampled after 0, 3, 6, 12, 24, and 48 h with five replications. Time 0 means the time point just before BPH infestation. Total RNA was extracted from the leaf sheaths and then converted into cDNA using a PrimeScript 1^st^ cDNA synthesis kit (TaKaRa, Japan). The expression of *BPH18* was evaluated by TaqMan qRT-PCR using ABI7900HT machine (Applied Biosystems, USA). The expression level in the samples was quantified relative to the first replicate of Time 0. To investigate *BPH18* expression in different tissues, we extracted total RNAs from the leaf sheath, leaf, and root of five-leaf-stage plants. The expression level in the samples was quantified relative to the first replicate of root. A genomic DNA fragment (2,340 bp to 1 bp from the translation start site) containing the promoter region of *BPH18* was amplified by PCR using primers attB-BPH18-pro-F and attB-BPH18-pro-R. This fragment was inserted into the upstream of the *beta-glucuronidase (GUS*) coding region in the pBGWFS7 binary vector. Transgenic plants carrying the above construct were generated in Dongjin cultivar background.

### Subcellular localization

The full-length *BPH18* coding region (3.7 kb) without a stop codon was amplified with primers BPH18-loc-F and BPH18-loc-R from the IR65482-7-216-1-2 cDNA. Finally, the fragment was cloned into the downstream of the *ZmUbi1* promoter and in frame with *GFP* in the pGA3452 vector and with *RFP* in the pGA3574 vector, respectively. Protoplasts were prepared from leaves of rice seedlings and the rice Oc cell line (suspension culture) which was derived from roots of rice seedlings. The constructs were co-transformed into protoplast through electroporation with several markers, including the ER marker (Bip:RFP), *cis*-Golgi marker (ManI:RFP), *trans*-Golgi network marker (N-ST:RFP), and prevacuolar compartments (PVC) marker (GFP:SYP21). After incubation, the protoplasts were observed under confocal laser-scanning microscopy (LSM 510 META, Zeiss, Germany) and images were obtained using Zeiss LSM Image Browser. The experiments were done at least twice for each marker and the representative images were taken for publication.

### BPH resistance mechanism analysis

To test the antibiosis effect, we grew T_1_ plants of the two *BPH18* transgenic lines, TC1 and TG7, with their susceptible wild-type variety Dongjin and *BPH18* donor. Twelve four-week-old seedlings of each line were transferred into test tubes with water. Around 7-11 BPH insects of second- or third-instar nymphs were put into each test tube. When the Dongjin seedlings began to die, both live and dead BPH insects were counted and the BPH survival rates were calculated for each test tube. The BPH resistance score of the rice seedlings in each test tube was evaluated according to a method described by Huang *et al*.^53^ as follows; 0 None of the leaves shrank and the plant was healthy, (1) One leaf was yellowing, (3) One to two leaves were yellowing or one leaf shrank, (5) One to two leaves shrank or one leaf shriveled, (7) Three to four leaves shrank or two to four leaves shriveled, the plant was still alive, (9) The plant died. To test the antixenosis effect of *BPH18*, TC1, TG7, and the *BPH18* donor line (IR65482) were compared with the susceptible variety Dongjin. We transplanted five four-week-old seedlings of the test lines and Dongjin into a 20-cm pot, placing rice seedlings along the circumference of the pot, alternatively. A 5-cm Petri dish was placed at the center of the pot where BPH nymphs were released, and the pot was covered with a light-transmitting mesh.

Two replications were done for each experiment. The number of hoppers that had settled on each plant was recorded at 3, 6, 24, 48 and 96 h after infestation.

### Development of NILs, its BPH bioassay, and qRT-PCR of defense-related genes

A BPH susceptible indica rice variety, IR24 as a recurrent parent, was crossed with IR65482-7-216-1-2 (*BPH18* donor) and ADR52 (*BPH26* donor), respectively. The F_1_ plants were backcrossed to the recurrent parent. The BC_1_F_1_ plants were screened with known linked markers to select those plants that contain the resistance gene from the donor parent. The selected plants carrying the target gene were used in the next backcrossing cycle. This procedure was repeated through the 3^rd^ backcross. The BC_3_F_1_ plants having the target gene were selfed to produce the BC_3_F_2_ generation which were again selected and selfed until BC_3_F_5._ The bioassay was done by the MBST method^1^ during the dry and wet seasons of 2014 at the IRRI, Philippines. Seedlings at the three-leaf stage were infested with second- or third-instar nymphs at a density of 10–12 nymphs per seedling, done in two replications. The percent of plant survival was observed one week after infestation or once the susceptible check was dead. For qRT-PCR of defense-related genes, seven-day-old seedling plants were infested with the Nueva Ecija BPH population. Leaf sheaths from three plants per line were collected before (0 h) and after BPH infestation (8, 24, 48, 72 h). Total RNAs were extracted using TRIzol reagent (Life technologies, USA) and genomic DNAs were removed by treatment of DNase I (TURBO DNA-free kit, Life technologies), then first strand cDNAs were synthesized by ImProm-II Reverse Transcription system (Promega, USA). Real-time PCRs were performed using SYBR Select Master Mix (Life technologies) with ABI7500 machine (Applied Biosystems). The primer sequences for the target genes were same with the previous reports by Du *et al*.^5^ and Wang *et al*.^7^. *OsAct1* gene was used as an internal control and the relative expression level was calculated based on the *ΔΔ*Ct method. Each data point represents the mean value of three biological replications.

### Whole genome sequencing and data analysis

Whole genome sequencing of NIL-*BPH18*, NIL-*BPH26*, and IR24 were conducted by Illumina Hiseq2500 platform at 30X coverage depth (pair-end 125 bp sequencing with average 500 bp insert library). *De novo* assembly was done by SOAPdenovo2 software^57^ with *k*-mer 59 after filtering the raw reads. The *BPH18*/*BPH26* gene with the surrounding regions was identified through pairwise sequence alignment between the newly created scaffolds and indica reference genome (93–11). The raw sequence reads were aligned against 93–11 reference genome using BWA software^58^, resulting in SAM files. Using SAMtools^59^, the SAM files were converted to BAM files and the consensus sequences were extracted. Finally the chromosomes were formed after comparing the reference based assembly and *de novo* assembly. The dot plot alignment of chromosome 12 was visualized using Mummer software^60^.

## Additional Information

**Accession codes:** The *BPH18* sequences from IR65482-7-216-1-2 (accession no. KF890252) and Junam (accession no. KJ850252) were deposited in the NCBI GenBank database.

**How to cite this article**: Ji, H. *et al*. Map-based Cloning and Characterization of the *BPH18* Gene from Wild Rice Conferring Resistance to Brown Planthopper (BPH) Insect Pest. *Sci. Rep.* **6**, 34376; doi: 10.1038/srep34376 (2016).

## Supplementary Material

Supplementary Information
