# Erratum: Map-based Cloning and Characterization of the *BPH18* Gene from Wild Rice Conferring Resistance to Brown Planthopper (BPH) Insect Pest

**DOI:** 10.1038/srep36688

**Published:** 2016-11-11

**Authors:** Hyeonso Ji, Sung-Ryul Kim, Yul-Ho Kim, Jung-Pil Suh, Hyang-Mi Park, Nese Sreenivasulu, Gopal Misra, Suk-Man Kim, Sherry Lou Hechanova, Hakbum Kim, Gang-Seob Lee, Ung-Han Yoon, Tae-Ho Kim, Hyemin Lim, Suk-Chul Suh, Jungil Yang, Gynheung An, Kshirod K. Jena

Scientific Reports 6: Article number: 3437610.1038/srep34376; published online: 09 29 2016; updated: 11 11 2016

In this Article, Jungil Yang and Gynheung An are incorrectly affiliated with “Division of Tree Breeding, National Institute of Forest Science Institute, Suwon, Korea”. In addition, Hyemin Lim is incorrectly afilliated with “Department of Plant Molecular Systems Biotechnology and Crop Biotech Institute, Kyung Hee University, Yongin, Korea”. The correction affiliations are listed below:

Jungil Yang

Department of Plant Molecular Systems Biotechnology and Crop Biotech Institute, Kyung Hee University, Yongin, Korea.

Gynheung An

Department of Plant Molecular Systems Biotechnology and Crop Biotech Institute, Kyung Hee University, Yongin, Korea.

Hyemin Lim

Division of Tree Breeding, National Institute of Forest Science Institute, Suwon, Korea.

