# Supplementary material for: Map-based Cloning and Characterization of the BPH18 Gene from Wild Rice Conferring Resistance to Brown Planthopper (BPH) Insect Pest
Source: Sci Rep. 2016 Sep 29;6:34376. doi: 10.1038/srep34376 (PMC5041133; doi:10.1038/srep34376)
Supplement: Supplementary Information [file srep34376-s1.pdf]

Map-based Cloning and Characterization of the *BPH18* Gene from Wild Rice Conferring Resistance to Brown Planthopper (BPH) Insect Pest

Hyeonso Ji[1], Sung-Ryul Kim[2], Yul-Ho Kim[3], Jung-Pil Suh[3], Hyang-Mi Park[3], Nese Sreenivasulu[2], Gopal Misra[2], Suk-Man Kim[4], Sherry Lou Hechanova[2], Hakbum Kim[3], Gang-Seob Lee[1], Ung-Han Yoon[1], Tae-Ho Kim[1], Hyemin Lim[1], Suk-Chul Suh[1], Jungil Yang[5], Gynheung An[5] and Kshirod K. Jena[2,*]

[1]Department of Agricultural Biotechnology, National Institute of Agricultural Sciences, Jeonju, Korea; [2]Plant Breeding Division, International Rice Research Institute (IRRI), Metro Manila, Philippines; [3]National Institute of Crop Science, Suwon, Korea; [4]IRRI-Korea Office, National Institute of Crop Science, Rural Development Administration, Jeonju, Korea; [5]Department of Plant Molecular Systems Biotechnology and Crop Biotech Institute, Kyung Hee University, Yongin, Korea

[*]**Author for correspondence:**

Kshirod K. Jena

Tel: +63 2 580 5600

Email: k.jena@irri.org

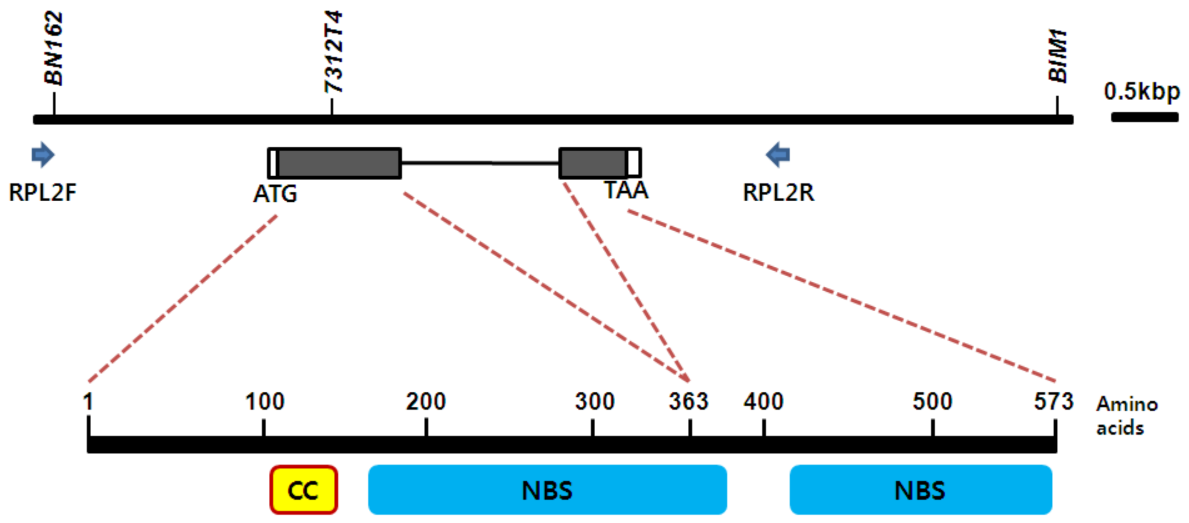

Supplementary Figure S1. The structure of the *LOC_Os12g37290* in IR65482 line and its encoding protein. Filled boxes indicate coding sequence and empty boxes indicate 5′UTR and 3′UTR. The line between boxes is intron. The RPL2F and RPL2R primers were used for amplification of the whole genomic region of the *LOC_Os12g37290* through long PCR.

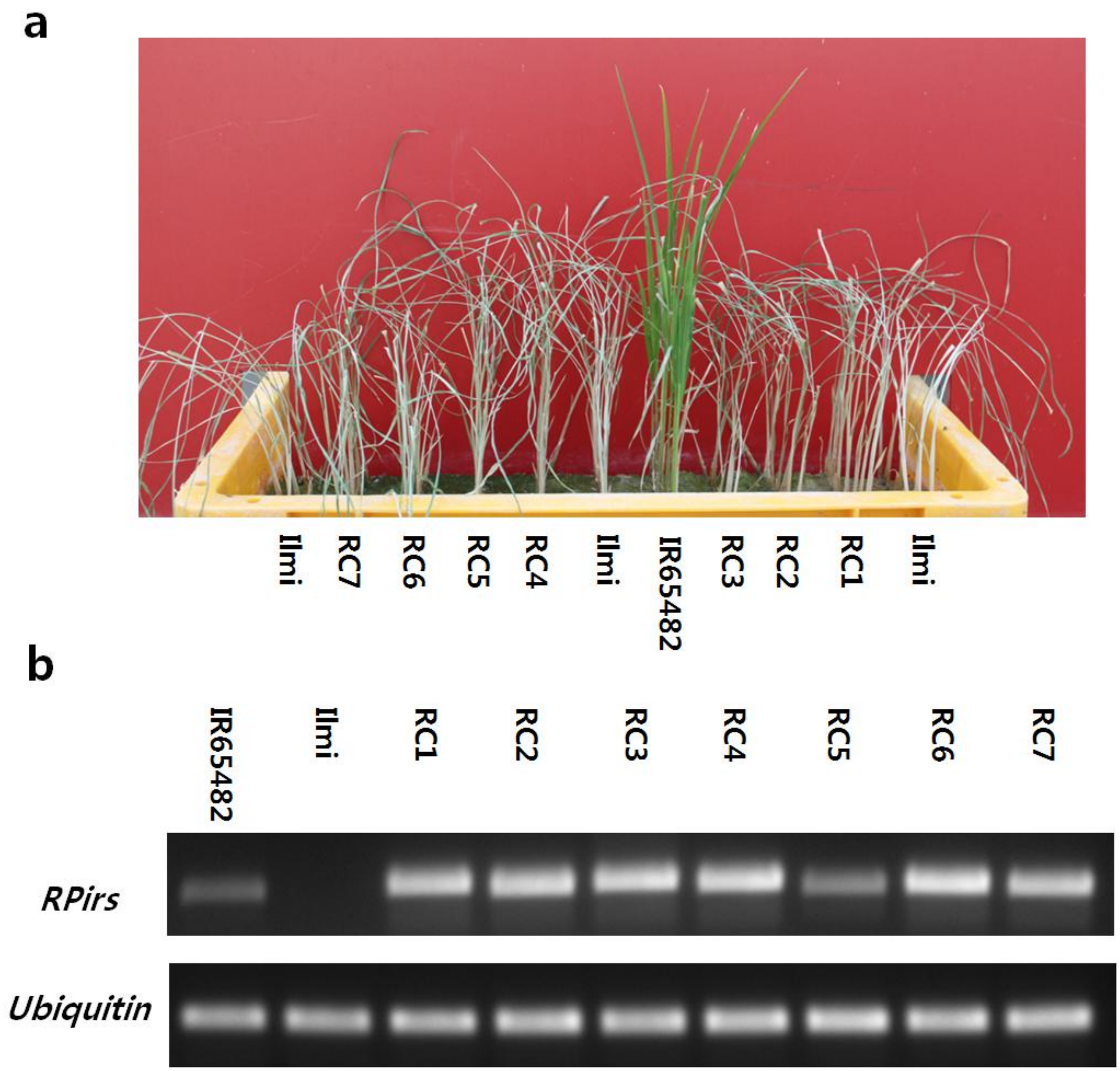

Supplementary Figure S2. Complementation test of the *LOC_Os12g37290*. (**a**) BPH bioassay of the transgenic lines harboring *LOC_Os12g37290* of IR65482. Ilmi, susceptible wild-type japonica variety; IR65482, resistant parental line; RC1-7, independent transgenic lines harboring the full *LOC_Os12g37290* genomic region (6.4kb), including its promoter and terminator. (**b**) RT-PCR analysis of the *LOC_Os12g37290* gene in transgenic T_1 lines. The RT-PCR primers (RPirs) were specifically designed to amplify the *LOC_Os12g37290* of the resistant parental line, IR65482.

MEATAVSIGRSVLKGALGFAKSTLVEEVSLQLGVQRDQAFIRDELEMMNSFLMAANDEKDDNKVVRT

WVKQVRDVAYDVEDCLQDFAVRLGGKSSTWWLSP***HTLWERRRIAKQMKELRGKVEDVSQRNMR***

YQLIKDSKPTVATNVTPSSTARATMSGAQEERWQ

CC

165

P-loop RNBS-A

HEKAIDHLVRLVKTKVDERRVIAVW**GTSGDIREM**SIVGGAYDHLKRNNK**FECCAWVNLMHPL**NP

TKLLQTIVRQFYVRSLQEAGKATPSCQILSSMLIKEDQGLGFRVLRSMLMKEDHLNDEFNKYLSDKCY

Walker B RNBS-B

LIVLNDLSTAEEWKQIKMLFPDNKK**GSRIIVFTQ**QVEVASFCARTEEVAPEHMQLFADQTLYAFHCK

GAKDGVDSMEDSSNLNEDTTYNAVEGKSLTCTYSMVTAFKESEIVG

NBS

410

P-loop RNBS-A

RVDEIKEIIELISKGSQQLEKISVW**GMGGIGKT**TLIQNVYRSEKVKKM**FDKHACVTIMRPF**NLNDLL

Walker B RNBS-B

MSLVRQLEDSKTSGGKELVSILEGKKY**LIVLDDV**LFTTEWDAIESYFPATET**GSRIIITTR**HESIAKHCSG

RNBS-C GLPL

DQQGK**MYQLNRLGDNDAKNLFAKKVF**KESVNLDQQDLELIKEAKPILKKCN**GLPL**AIVTIGGFLAS

RNBS-D

RPKTTLEWRKLNEHISAELETNPGLEAIRAVLNISYDGLPYHLKSC**FLYLSIFP**EDGKISRKRLVRRWCAE

MHD

GYSRELWDKSAEEIANNYFFELIDRSMILPTQNSTYSSRGADSCQ**IHD**IMREIAILKSKEENLVLRLEGG

PRLYNHDTVRHISITNSSEDWETDVGELKTTVDMSRIRSLTVFGMWRPFFISDKMQL

NBS

812

LQVLDLEDTNGVYDHHIKQIGK

LLHLRYLSLRGCRDITYLPDS

LGNLRQLETLDVRGTCILRLQKT

IINLRKLKYLRAVPNLSDPYEDIAEK

LPELIRNRLCISATV

LLALCVLCSASAICKFDVGISTRD

LCTLCCCSILPAIAMRLDGNGVVAPRG

LRRLTALHTLGVVDISWQPSILQDIKR

LIQLRKLGVSGVNKKNSKKF

LSALVALSRLESLSLISKGKPGLWGCLDADEKFSPPKN

LKTLKLQGNLVGLPKWIGQ

LNNLVKLKLSETGLKDHDAAIQV

LGKLRNLTILCLLGKSFHSLEGGELNFSEGSFKS

LVVLELDLSGSKCVKFQQGAFHN

LELLELDCEPRELSAGHIEEIKTKFSG

LEFLPRIKEVRLQGYFYGSCDTRK

LKEDLLAQLSENPKKPILKTSR

LRR

Supplementary Figure S3. Analysis of the conserved domains of BPH18. The coiled-coil motif sequence was underlined and italicized. The conserved sequence motifs of NBS domains were underlined with their motif names.

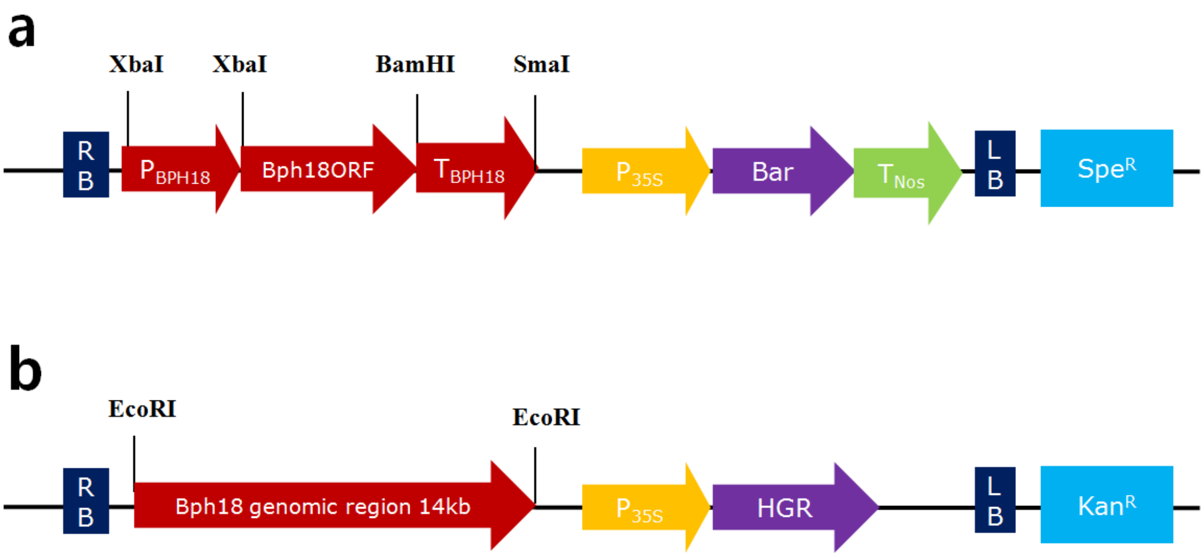

Supplementary Figure S4. Schematic representation of complementation vector constructs for *BPH18*. (**a**) The *BPH18* ORF fused with the *BPH18* promoter and terminator was cloned into the plant expression vector, pPZP. The restriction enzyme sites used in cloning the *BPH18* promoter, *BPH18* ORF, and *BPH18* terminator are shown. (**b**) The *BPH18* genomic region, including the promoter and terminator of the resistance donor line was cloned into the plant expression vector, pCAMBIA1300.

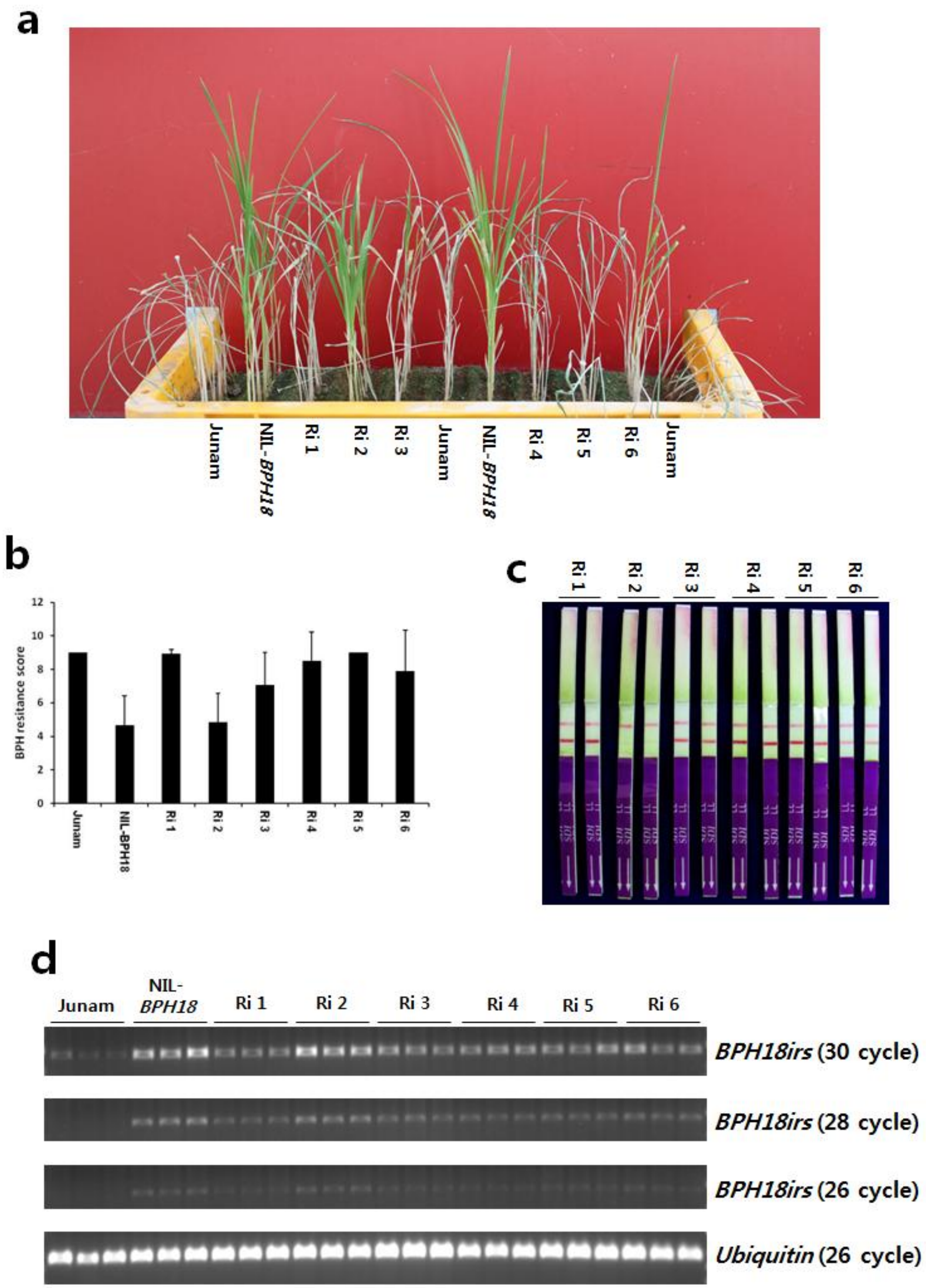

Supplementary Figure S5. Suppression of *BPH18* using RNAi construct in the BPH resistant line NIL-*BPH18*. (**a**) BPH bioassay of the *BPH18* RNAi transgenic lines. Junam, wild type susceptible variety; NIL-*BPH18*, a NIL line harboring a *BPH18* gene with Junam background. Ri1-6: *BPH18* RNAi transgenic lines derived from the NIL-*BPH18* line. (**b**) BPH-

resistance scores of the *BPH18* RNAi transgenic plants at the seedling stage. Lower scores indicate a higher resistance to the insect. Data are means ± standard deviation. (**c**) *bar*-strip test of RNAi transgenic lines. Two bands indicate the existence of a *trans*-gene and one band indicate the absence of a *trans*-gene. (**d**) RT-PCR analysis of *BPH18* gene in the RNAi transgenic lines. The *BPH18* irs primer pair were specifically designed to amplify the *BPH18* gene of the resistant parental line, IR65482. *Ubiquitin* was used as a reference gene. The lines (Ri1, Ri3-6) having RNAi construct showed suppressed expression of the *BPH18* gene. However, the Ri2 line lacked RNAi construct showed a similar expression level with its original line, NIL-*BPH18*.

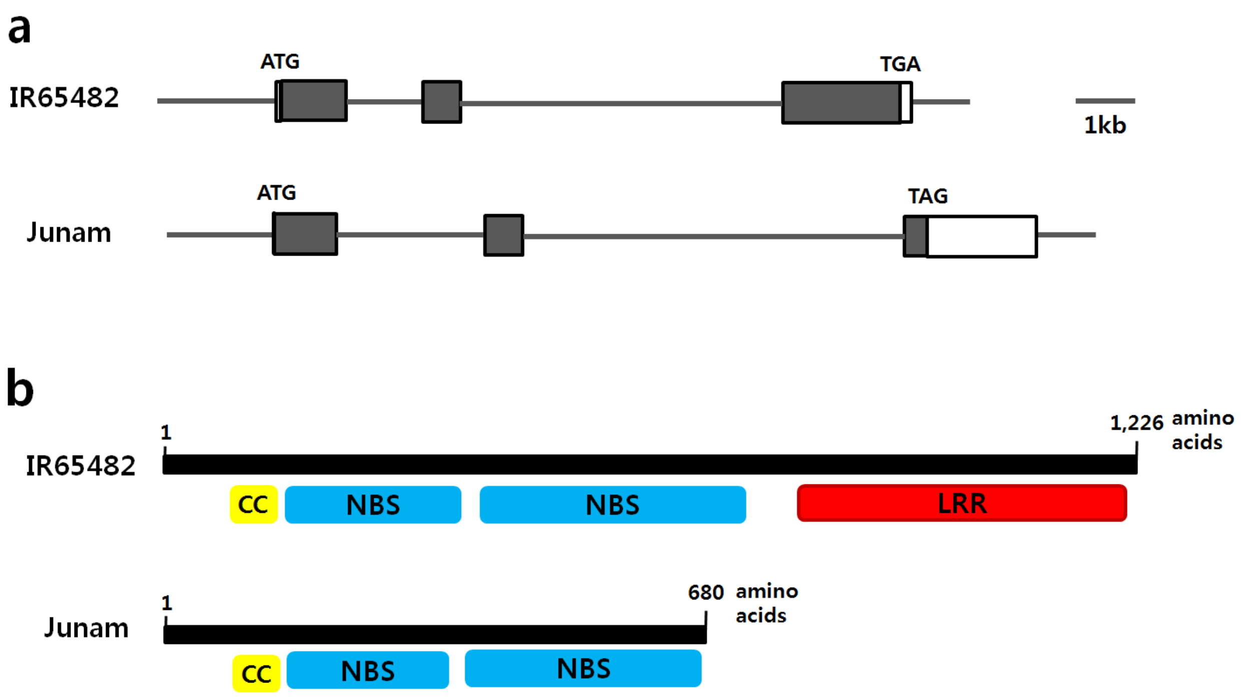

Supplementary Figure S6. Comparison of the gene structure and the deduced protein structure of *BPH18* between the resistance donor line and susceptible parental variety, Junam. (**a**) Genomic structures of the *BPH18*. Rectangular boxes are exons, whereas lines between them are introns. Empty boxes are 5′ and 3′ UTRs and filled boxes are coding sequences. The translation start codon (ATG) and stop codon (TGA and TAG) are shown. (**b**) Protein domain structures of BPH18. The CC, NBS, and LRR domains are shown under the bar representing the length of the proteins.

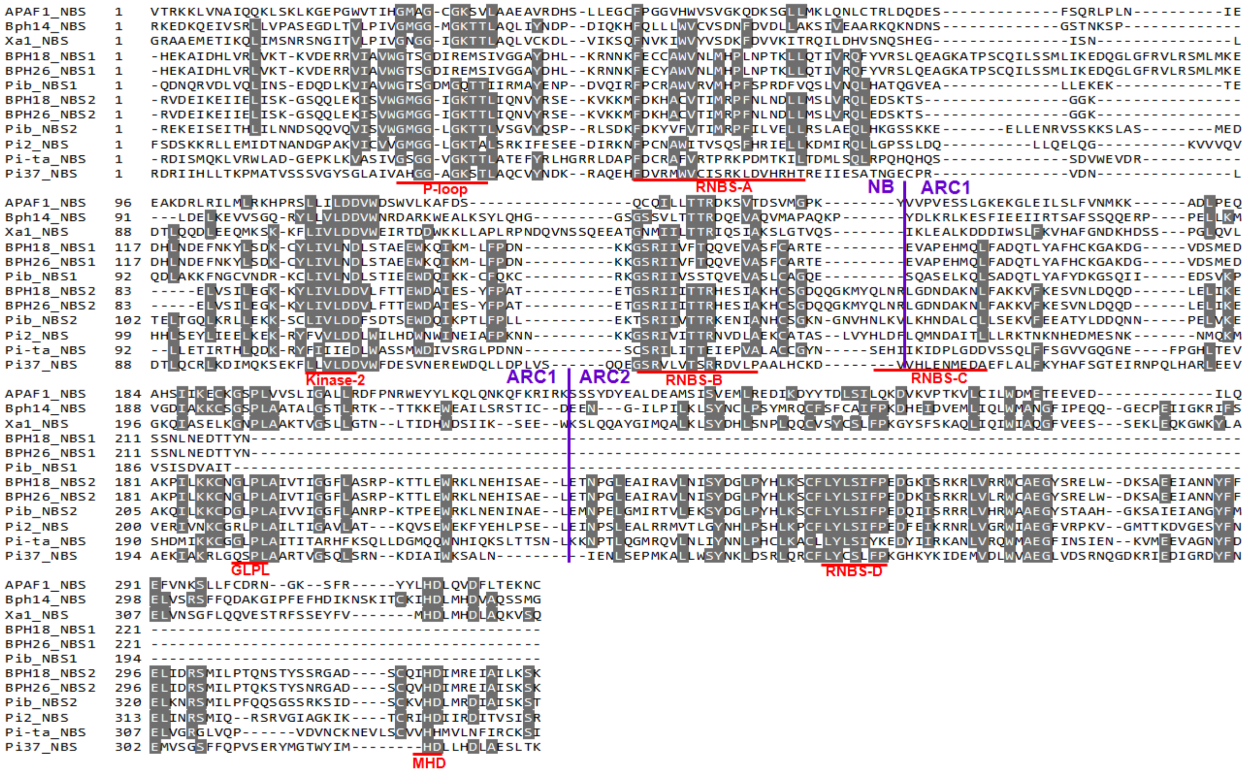

Supplementary Figure S7. Multiple-sequence alignment of the NB, ARC1, and ARC2 sub-domains of NBS-LRR R proteins and APAF-1. Domain borders are indicated as vertical violet lines. Motifs are annotated as horizontal red lines below the aligned sequences. The identical amino acids were shaded (threshold = 50%).

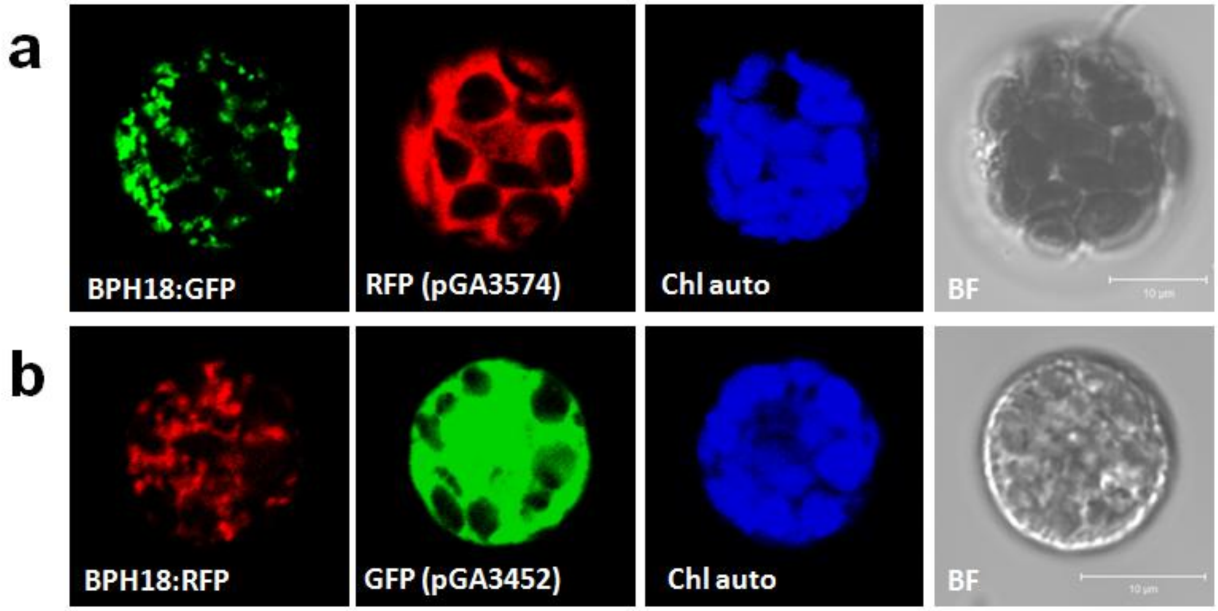

Supplementary Figure S8. Transient expression of the BPH18 protein in the rice protoplast derived from seedling shoots. The *pZmUbi1*::*BPH18*:*GFP* construct (**a**) and the *pZmUbi1*::*BPH18*:*RFP* construct (**b**) was co-transfected with the *pZmUbi1*::*RFP* vector (pGA3574) and the *pZmUbi1*::*GFP* vector (pGA3452), respectively. The fluorescence signals from GFP, RFP, and chlorophyll were detected from the protoplasts under confocal microscopy. Chl auto, chlorophyll auto-fluorescence; BF, bright field; scale bar = 10 µm.

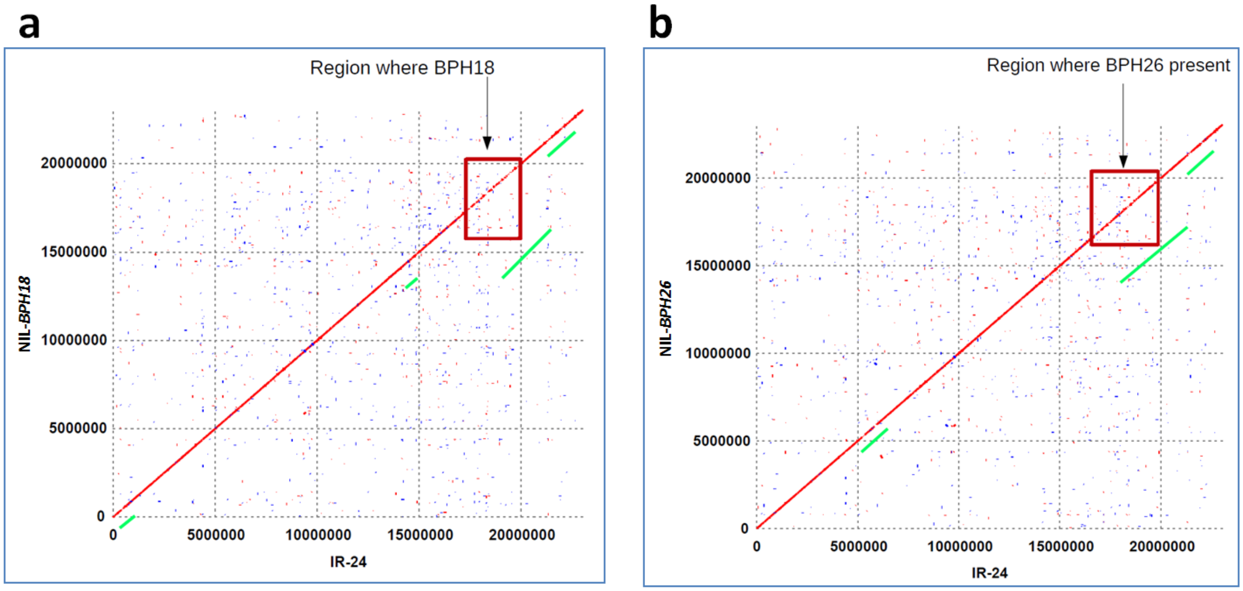

Supplementary Figure S9. Dot plot alignment of chromosome 12 using Mummer. Green bar represents the chromosome segment which is derived from the donor lines. (**a**) Alignment between IR24 and NIL-*BPH18*. (**b**) Alignment between IR24 and NIL-*BPH26*.

```
BPH18   1      ATGGAGGCCACGGCGGTGAGCATTGGCAGGTCCGTGCTGAAGGGAGCGCTTGGCTTCGCC
BPH26   1      ATGGAGGCCACGGCGGTGAGCATTGGCAGGTCCGTGCTGAAGGGAGCGCTTGGCTTCGCC

BPH18   61     AAATCCACCTTGGTGGAGGAGGTTTCCCTGCAGCTCGGCGTCCAGCGTGACCAGGCGTTC
BPH26   61     AAATCCACCTTGGTGGAGGAGGTTTCCCTGCAGCTCGGCGTCCAGCGTGACCAGGCGTTC

BPH18   121    ATCAGGGACGAGCTGGAGATGATGAACTCCTTCCTGATGGCCGCCAATGATGAGAAAGAT
BPH26   121    ATCAGGGACGAGCTGGAGATGATGAACTCCTTCCTGATGGCCGCCAATGATGAGAAAGAT

BPH18   181    GACAACAAGGTGGTAAGGACCTGGGTGAAGCAGGTCCGCGACGTGGCCTACGACGTTGAG
BPH26   181    GACAACAAGGTGGTAAGGACCTGGGTGAAGCAGGTCCGCGACGTGGCCTACGACGTTGAG

BPH18   241    GACTGCCTCCAGGACTTCGCCGTCCGCTTGGGGGGGAAGAGTTCAACCTGGTGGCTCAGC
BPH26   241    GACTGCCTCCAGGACTTCGCCGTCCGCTTGGGGGGGAAGAGTTCAACCTGGTGGCTCAGC

BPH18   301    CCTCACACGCTGTGGGAGCGGCGCCGCATCGCCAAGCAGATGAAGGAGCTGAGGGGCAAG
BPH26   301    CCTCACACGCTGTGGGAGCGGCGCCGCATCGCCAAGCAGATGAAGGAGCTGAGGGGCAAG

BPH18   361    GTTGAGGATGTCAGCCAGAGGAACATGCGCTATCAACTCATCAAGGACTCCAAGCCTACT
BPH26   361    GTTGAGGATGTCAGCCAGAGGAACATGCGCTATCAACTCATCAAGGGCTCCAAGCCTACT

BPH18   421    GTAGCTACCAATGTCACACCCAGCAGCACTGCCCGTGCGACCATGTCTGGCGCGCAGGAA
BPH26   421    GTAGCTACCAATGTCACACCCAGCAGCACTGCCCGTGCGACCATGTCTGGCGCGCATGAA

BPH18   481    GAACGGTGGCAGCATGAGAAGGCAATAGATCATCTGGTTCGGCTGGTCAAAACCAAAGTC
BPH26   481    GAACGGTGGCAGCATGAGAAGGCAATAGATCATCTGGTTCGGCTGGTCAAAACCAAAGTC

BPH18   541    GATGAACGTAGAGTGATCGCGGTGTGGGGAACAAGTGGTGATATCAGGGAAATGTCCATC
BPH26   541    GATGAACGTAGAGTGATCGCGGTGTGGGGAACAAGTGGTGATATCAGGGAAATGTCCATC

BPH18   601    GTTGGAGGGGCCTATGATCATCTCAAGAGAAACAACAAGTTTGAGTGCTGTGCCTGGGTT
BPH26   601    GTTGGAGGGGCCTATGATCATCTCAAGAGAAACAACAAGTTTGAGTGCTATGCCTGGGTT

BPH18   661    AATTTGATGCATCCTCTGAACCCAACAAAGCTCCTGCAAACCATTGTTAGGCAATTCTAT
BPH26   661    AATTTGATGCATCCTCTGAACCCAACAAAGCTCCTGCAAACCATTGTTAGGCAATTCTAT

BPH18   721    GTAAGATCTCTTCAGGAGGCTGGCAAAGCAACTCCCTCGTGTCAAATTCTGAGTAGCATG
BPH26   721    GTAAGATCTCTTCAGGAGGCTGGCAAAGCAACTCCGTCGTGTCAAATTCTGAGTAGCATG

BPH18   781    TTGATAAAGGAAGATCAGGGTTTGGGGTTTAGGGTTCTGAGGAGCATGCTGATGAAGGAA
BPH26   781    TTGATAAAGGAAGATCAGGGTTTGGGGTTTAGGGTTCTGAGGAGCATGCTGATGAAGGAA

BPH18   841    GATCATTTGAATGATGAGTTCAATAAATATTTGAGTGACAAGTGCTACCTCATTGTGCTT
BPH26   841    GATCATTTGAATGATGAGTTCAATAAATATTTGAGTGACAAGTGCTACCTCATTGTGCTT

BPH18   901    AATGACCTATCGACCGCTGAAGAGTGGAAGCAAATTAAAATGCTCTTCCCAGACAACAAG
BPH26   901    AATGACCTATCGACCGCTGAAGAGTGGAAGCAAATTAAAATGCTCTTCCCAGACAACAAG

BPH18   961    AAAGGAAGCCGAATCATAGTGTTCACACAACAAGTTGAAGTTGCAAGCTTTTGCGCTAGG
BPH26   961    AAAGGAAGCCGAATCATAGTGTTCACACAACAAGTTGAAGTTGCAAGCTTTTGCGCTAGG

BPH18   1021   ACAGAGGAGGTGGCACCCGAGCACATGCAGTTGTTTGCTGATCAGACTCTTTATGCTTTT
BPH26   1021   ACAGAGGAGGTGGCACCCGAGCACATGCAGTTGTTTGCTGATCAGACTCTTTATGCTTTT

BPH18   1081   CACTGCAAGGTAATACTAAAAAAAAA-TCCTCAAACATGCACTGAAAAAGAAATATTCTA
BPH26   1081   CACTGCAAGGTAATACTAAAAAAAAAATCCTCAAACATGCACTGAAAAAGAAATATTCTA
```

BPH18	1140	TATATCATCATCCATTGAAAGCATACTCTTCTCATTCAACTTGTCTGCTAATTAAATCAA
BPH26	1141	TATATCATCATCCATTGAAAGCATACTCTTCTCATTCAACTTGTCTGCTAATTAAATCAA
BPH18	1200	TGTTTTAGCTATGACATTATGTACACAATAAACTAACACTAATTAATACCACATAGCTCT
BPH26	1201	TGTTTTAGCTATGACATTATGTACACAATAAACTAACACTAATTAATACCACATAGCTCT
BPH18	1260	GTCTCTTTCATCTCTAAGAGCATCTCCAACAGACTATATATCGTACTCTCTGTAGTTATA
BPH26	1261	GTCTCTTTCATCTCTAAGAGCATCTCCAACAAACTATATATCGTACTCTCTGTAGTTATA
BPH18	1320	ATTCAGCAATTCTCTTTAAAAAAATAACAGCTCCAATAAATCACCCTATCTTAGTCCCTA
BPH26	1321	ATTCAGCAATTCTCTTTAAAAAAATAACAGCTCCAATAAATCACCCTATCTTAGTCCCTA
BPH18	1380	AATTTTAGGTCCTCCCCATAGTGAGTATAATCGACCCCAAATTTTGGGTGCCCCTGTCCC
BPH26	1381	AATTTTAGGTCCTCCCCATAGTGAGTATAATCGACCCCAAATTTTGGGTGCCCCTGTCCC
BPH18	1440	ACTCCCTATGCCAGCCAAGACTCGCTGATCGTTATCGTTTTCCATGCATCCTCCACCTTC
BPH26	1441	ACTCCCTATGCCAGCCAAGACTCGCTGATCGTTATCGTTTTCCATGCATCCTCCACCTTC
BPH18	1500	CTCTCCTCCCCACATCCCTCGTCTTGCTTGGCCTTCGCCTTTCGCCTCCTGCGACCACCA
BPH26	1501	CTCTCCTCCCCACATCCCTCGTCTTGCTTGGCCTTCGCCTTTCGCCTCCTGCGACCACCA
BPH18	1560	CTTAGCTGGCCATTGCTCTCTTCACCATCAGGCTCCAACCTCTGTTGAAAGCATATTTCA
BPH26	1561	CTTAGCTGGCCATTGCTCTCTTCACCATCAGGCTCCAACCTCTGTTGAAAGCATATTTCA
BPH18	1620	TTTTGCATGTTGGAACGACAACTCTATCTTAAACTTACTGCTTAAAAGCCAACAATTTGT
BPH26	1621	TTTTGCATGTTGGAACGACAACTCTATCTTAAACTTACTGCTTAAAAGCCAACAATTTGT
BPH18	1680	AAGCTATATAGGACAACATGCCAGCTTATAGGCATACTGCTAAGCCTGTGATTAATTTGC
BPH26	1681	AAGCTATATAGGACAACATGCCAGCTTATAGGCATACTGCTAAGCCTGTGATTAATTTGC
BPH18	1740	TTGTTTTTAGTATTATGCAATACGGTAGTACTTTGAGCTCAACTTTAACCTATCCTGCAT
BPH26	1741	TTGTTTTTAGTATTATGCAATACGGTAGTACTTTGAGCTCAACTTTAACCTATCCTGCAT
BPH18	1800	AACACATTTTTAGAGGATTTTGAAGTTCACAAATGTGTAGATAGATGCATTTGCACTTAT
BPH26	1801	AACACATTTTTAGAGGATTTTGAAGTTCACAAATGTGTAGATAGATGCATTTGCACTTAT
BPH18	1860	CTGAAAATTCCCATATGGATACTTGATTATTATGTACTATAATCAGGACCCCACCAAATT
BPH26	1861	CTGAAAATTCCCATATGGATACTTGATTATTATGTACTATAATCAGGACCCCACCAAATT
BPH18	1920	TACAGATTGATAAGTCTTTTAATTGATCGGGCAAGTACTATATGGAGTAACACTTATGGG
BPH26	1921	TACAGATTGATAAGTCTTTTAATTGATCGGGCAAGTACTATATGGAGTAACACTTATGGG
BPH18	1980	TCGCAAGGCATTTAAAAATTAAGTAGTTCAATTGAGATTGTCTTTTCACTAAAGAATGGT
BPH26	1981	TCGCAAGGCATTTAAAAATTAAGTAGTTCAATTGAGATTGTCTTTTCACTAAAGAATGGT
BPH18	2040	AACCCATTTATACATTTACAAGAGATTTGTCTTGGGAAACAACTAAGTATTATCATAGGA
BPH26	2041	AACCCATTTATACATTTACAAGAGATTTGTCTTGGGAAACAAGTAAGTATTATCATAGGA
BPH18	2100	AAGTAGACAATTAGACATCATCATCTAATATTCTAAATAATAACTGTTCTGAAGCAGTTG
BPH26	2101	AAGTAGACAATTAGACATCATCATCTAATATTCTAAATAATAACTGTTCTGAAGCAGTTG
BPH18	2160	GCTACAATTTGTTGTTGTTGTTTCATTGAAAAGTTCTTGCAACCTTTTTTTTTTCCATTA
BPH26	2161	GCTACAATTTGTTGTTGTTGTTTCATTGAAAAGTTCTTGCAACCTTTTTTTTTTC-ATTA
BPH18	2220	AGGGCATGTACGATTCCTTTTTAACTCTCGTCTCGTCTTAAAATATAATGGTGCACACCT
BPH26	2220	AGGGCATGTATGATTCCTTTTTAACTCTCGTCTCGTCTTAAAATATAATGGTGCACACCT

```
BPH18  2280  CTCATGCGTGTTTAAGAAAAGAATATTGTTGATAATC-TTTTTTTTGTGTGTGTGCAGGG
BPH26  2280  CTCATGCGTGTTTAAGAAAAGAATATTGTTGATAATCATTTTTTTTGTGTGTGTGCAGGG

BPH18  2339  TGCTAAAGATGGAGTAGATTCAATGGAGGACTCATCTAACTTAAACGAAGACACTACATA
BPH26  2340  TGCTAAAGATGGAGTAGATTCAATGGAGGACTCATCTAACTTAAACGAAGACACTACATA

BPH18  2399  CAACGCTGTAGAAGGAAAGAGCCTCACTTGCACATATTCAATGGTAACTGCTTTCAAGGA
BPH26  2400  CAACGCTGTAGAAGGAAAGAGCCTCACTCGCACATATTCAATGGTAACTGCTTTCAAGGA

BPH18  2459  ATCTGAGATCGTTGGGCGAGTTGATGAAATAAAGGAGATTATTGAACTGATTTCAAAAGG
BPH26  2460  ATCTGAGATCGTTGGGCGAGTTGATGAAATAAAGGAGATTATTGAACTGATTTCAAAAGG

BPH18  2519  TAGCCAACAACTTGAGAAGATCTCAGTGTGGGGAATGGGTGGTATTGGGAAAACCACTCT
BPH26  2520  TAGCCAACAACTTGAGAAGATCTCAGTGTGGGGAATGGGTGGTATTGGGAAAACCACTCT

BPH18  2579  AATTCAAAATGTCTACCGAAGCGAAAAGGTTAAGAAGATGTTTGATAAGCATGCATGTGT
BPH26  2580  AATTCAAAATGTCTACCGAAGCGAAAAGGTTAAGAAGATGTTTGATAAGCATGCATGTGT

BPH18  2639  CACGATCATGCGCCCGTTCAATCTTAATGATCTTCTTATGAGCTTAGTTAGGCAACTAGA
BPH26  2640  CACGATCATGCGCCCGTTCAATCTTAATGATCTTCTTATGAGCTTAGTTAGGCAACTAGA

BPH18  2699  AGATTCAAAAACTTCTGGAGGAAAGGAGTTGGTTAGCATTTTAGAAGGAAAGAAATACTT
BPH26  2700  AGATTCAAAAACTTCTGGAGGAAAGGAGTTGGTTAGCATTTTAGAAGGAAAGAAATACTT

BPH18  2759  GATTGTTCTTGATGATGTATTATTCACAACAGAATGGGATGCTATAGAATCATATTTCCC
BPH26  2760  GATTGTTCTTGATGATGTATTATTCACAACAGAATGGGATGCTATAGAATCATATTTCCC

BPH18  2819  AGCAACGGAAACAGGAAGCCGGATCATAATAACCACAAGGCATGAAAGTATTGCTAAGCA
BPH26  2820  AGCAACGGAAACAGGAAGCCGGATCATAATAACCACAAGGCATGAAAGTATTGCTAAGCA

BPH18  2879  TTGTTCAGGGGATCAACAAGGAAAAATGTATCAACTCAATCGTCTAGGAGACAACGATGC
BPH26  2880  TTGTTCAGGGGATCAACAAGGCAAAATGTATCAACTCAATCGTCTAGGAGACAACGATGC

BPH18  2939  AAAGAACCTCTTTGCAAAGAAGGTAACTTAAAAGCATATATACTTCTCTCCATTGCTACG
BPH26  2940  AAAGAACCTCTTTGCAAAGAAGGTAACTTAAAAGCATATATACTTCTCTCCATTGCTACG

BPH18  2999  CACATTACTACTGCTACTTAGTCTAGCTAGCTACCTTTGGAAAGCGAAACGGCTACCTAT
BPH26  3000  CACATTACTACTGCTACTTAGTCTAGCTAGCTACCTTTGGAAAGCGAAACGGCTACCTAT

BPH18  3059  GAATCCAAACATAAATCATTTGAACTTCTACATAGAGATAATGAAGTGATTGTGTGATTT
BPH26  3060  GAATCCAAACATAAATCATTTGAACTTCTACATAGAGATAATGAAGTGATTGTGTGATTT

BPH18  3119  AATTATCGTTCATTTACACAAAATCAGAAAGGTCATGTGATATACTCATGGGTGATGGAG
BPH26  3120  AATTATCGTTCATTTACACAAAATCAGAAAGGTCATGTGATATACTCATGGGTGATGGAG

BPH18  3179  TTTACTACACCCTCTCAACTAGATGATGTCTTTCTTTGCAATAAATAAAGTATGTCCTAG
BPH26  3180  TTTACTACACCCTCTCAACTAGATGATGTCTTTCTTTGCAATAAATAAAGTATGTCCTAG

BPH18  3239  ATACATGCAATGAAACCTCAAATATGTTAAATTATCAATATATGAAAATGATATTGATAA
BPH26  3240  ATACATGCAATGAAACCTCAAATATGTTAAATTATCAATATATGAAAATGATATTGATAA

BPH18  3299  ACTTATGATGAAAATGCTTTAATATAGTTATATTTTAATTATTTATAAACATATAATAGA
BPH26  3300  ACTTATGATGAAAATGCTTTAATATAGTTATATTTCAATTATTTATAAACATATAATAGA

BPH18  3359  ATTGATGAACATATGCCTTAAAGACTGTATCAGTGTCCAAAATGTCATGTAAAAGAGAAT
BPH26  3360  ATTGATGAACATATGCCTTAAAGACTGTATCAGTGTCCAAAATGTCATGTAAAAGAGAAT
```

BPH18 3419 CCATATGTACACAATATACCAAGGGAAATAGAAGAAAGAATAAGAAAAACCATATTGAAG
BPH26 3420 CCATATGTACACAATATACCAAGGGAAATAGAAGAAAGAATAAGAAAAACCATATTGAAG

BPH18 3479 CAACGCTTGAAATGCAGGAATTTGAGAGTATTCATAATATTACTAACTCACAGTTCACTA
BPH26 3480 CAACGCTTGAAATGCAGGAATTTGAGAGTATTCATAATATAACTAACTCACAGTTCACTA

BPH18 3539 GGAACAGGAAAAATCCGCAAATTTTAAACAGGCCATTATATTTTCAGTTACAGGAAGTAT
BPH26 3540 GGAACAGGAAAAATCCGCAAATTTTAAACAGGCCATTATATTTTCAGTTACAGGAAGTAT

BPH18 3599 TATTTTAGCCGCTCTAGCATAAGATGGAGGGGGAAAACAGTTGGTAGCCAACTAGCTAGC
BPH26 3600 TATTTTAGCCGCTCTAGCATAAGATGGAGGGGGAAAACAGTTGGTAGCCAACTAGCTAGC

BPH18 3659 AGCAATTAAACCTCAGGGTTTTGAGGTAGCCTTACAGCCATTTGATAGCAGTAGACTCAT
BPH26 3660 AGCAATTAAACCTCAGGGTTTTGAGGTAGCCTTACAGCCATTTGATAGCAGTAGACTCAT

BPH18 3719 ACTCTAGGCCTTCTGGGTTATGATCCCATCCTATAATAATAAAATCCATGGTGCTATTTG
BPH26 3720 ACTCTAGGCCTTCTGGGTTATGATCCCATCCTATAATAATAAAATCCATGGTGCTATTTG

BPH18 3779 AAAATAAAGTTTCAATTCACGGATCTTTCACTCTATTTTACATTTAACAAAAAAAAA-CA
BPH26 3780 AAAATAAAATTTCAATTCACGGATCTTTCACTCTATTTTACATTTAACAAAAAAAAAACA

BPH18 3838 TCATTTACTCGTGAAAATACAATGACCTACCCTCTTCCAGGGTTTGCACCTATGTAAAGC
BPH26 3840 TCATTTACTCGTGAAAATACAATGACCTACCCTCTTCCAGAGTTTGCACCTATGTAAAGC

BPH18 3898 AATTAGTGGGGTTGTTCGATGATCTAATGCCCGAACGAAGACTTCGAATCCTTTTGTACA
BPH26 3900 AATTAGTGGGGTTGTTCGATGATCTAATGCCCGAACGAAGACTTCGAATCCTTTTGTACA

BPH18 3958 GATAAAGTGACGTCCCTTCATTCAAATGTCTGTTGCAAACAGGCACTTGTATCCATCTGA
BPH26 3960 GATAAAGTGACGTCCCTTCTTTCAAATGTCTGTTGCAAACAGGCACTTGTATCCATCTGA

BPH18 4018 TTATATCTTTGTCCATTGGAAACCTCTTTACCATCAGACACATGTGTAGTTAGTGAGCCG
BPH26 4020 TTATATCTTTGTCCATTGGAAACCTCTTTACCATCAGACACATGTGTAGTTAGTGAGCCG

BPH18 4078 TTCGCAGCAGAACAAGGCCAGCTATGCATGCTACCAACAACAA---ATATTTGTTACCAT
BPH26 4080 TTCGCAGCAGAACAAGGCCAGCTATGCATGCTACCAACAACAACAAATATTTGTTACCAT

BPH18 4135 ATGCTATTCATATGTAAACAATCACTTGTACCTTTTTTCCTAAAAAAACATAAAGTGAAT
BPH26 4140 ATGCTATTCATATGTAAACAATCACTTGTACCTTTTTTCCTAAAAAAACATAAAGTGAAT

BPH18 4195 TAGAATATAGATCACATTTTATTGCTGAAGTTTCACTTTGAACTAGGTAATTTTGCATTT
BPH26 4200 TAGAATATAGATCGCATTTTATTGCTGAAGTTTCACTTTGAACTAGGTAATTTTGCATTT

BPH18 4255 GTAGCAATTTCGACCACCCCAAACCATTATTTTCAGCACATCGGCTCTAGCGTGCTGATG
BPH26 4260 GTCGCAATTTCGACCACCCCAAACCATTATTTTCAGCACATCGGCTCTAGCGTGCTGATG

BPH18 4315 AACTATCTGAACAGTGTGCTAAGCCATATCTTTAATAGAGGAGTGATTGGACATGGCTGA
BPH26 4320 AACTATCTGAACAGTGTGCTAAACCATATCTTTAATAGAGGAGTGATTGGACATGGCTGA

BPH18 4375 AGAGGGAAGTTGAGGTGGTTTAAGGTGCAAAAAAGAATTGTAAGTAATCCAAAGTTAAAA
BPH26 4380 AGAGGGAAGTTGAGGTGGTTTAAGGTGCAAAAAAGAATTGTAAGTAATCCAAAGTTAAAA

BPH18 4435 GATCAGTCTGGTTCAAAGTGAAACTTTGCAATAAAAGATGATCCAAATTGTAATTCACTC
BPH26 4440 GATCAGTCTGGTTCAAAGTGAAACTTTGCAATAAAAGATGATCCAAATTGTAATTCACTC

BPH18 4495 AATTATGTTATAAATATTAAAAAATTAATTCCATATTATCTAATTGCATTACATCCTAAAT
BPH26 4500 AATTATGTTATAAATACAAAAAATTAATTCCATATTATCTAATTGCATTACATCCTAAAT

```
BPH18   4555   AAAAGCATGAAACTTTTATTTATTTCGGTCTAACCATTTAAAAACTATTGGTGGTTGATA
BPH26   4560   AAAAGCATGAAACTTTTATTTATTTCGGTCTAACCATTTAAAAACTATTGGTGGTTGATA

BPH18   4615   TTAAAAAGTAAAGTTTACAATTGCAAGTATTTGCGCACATAGTGTAGTATCTTATTATAT
BPH26   4620   TTAAAAAGTAAAGCTTACAATTGCAAGTATTTGCGCACATAGTGTAGTATCTTATTATAT

BPH18   4675   ATATAT------TATAGCCACTAACACACACACACACACAACCCCTACAAATGTACTAAA
BPH26   4680   ATATATATATATTATAACCACTAACACACACACACAC--AACCCCTACAAATGTACTAAA

BPH18   4729   TGCTAGATACTCTTTATTTACATACCAACATAAAAAATCATATGTACACAACCGTACGTA
BPH26   4738   TGCTAGATACTCTTTATTTACATACCAACATCACAAATCATATGTACACAACCGTACGTA

BPH18   4789   CGTACTTACAGATAAATAACAGACATACAGGTCTCACCAGAGGTATGTGGTGTGGTAGAG
BPH26   4798   CGTACTTACAGATAAATAACAGACATACAGGTCTCATCAGAGGTATGTGGTGTGGTAGAG

BPH18   4849   TTGTGCATGAATGCATAACTCTACCCATTAGGATCAAATCATAGTATTTACAAATATTAC
BPH26   4858   TTGTGCATGAATGCATAACTCTACCCATTAGGATCAAATCATAGTGTTTACAAATATTAC

BPH18   4909   ATACATGGGTAGTGGAGTTTAGTGATGTTAGATACCACTGGCTTCTATCTCTCTTTAAGC
BPH26   4918   ATACATGGGTAGTGGAGTTTAGTGATGTTAGATACCACTGGCTTCTATCTCTCTTTAAGC

BPH18   4969   GTGTGTTAAGAGAGTGTACTTGCGTGCACGCATATGCTTAATGTGCATCGAGGTCTTCAG
BPH26   4978   GTGTGTTAAGAGAGTGTACTTGCGTGCGCGCATATGCTTAATGTGCATCGAGGTCTTCAG

BPH18   5029   TGTAATAGAAGAAAAAAA-TAAAAAATAAGCAATTTACAAAGAAAATAAATAGGGAATCA
BPH26   5038   TGTAATAGAAGAAAAAAAATAAAAAATAAGCAATTTACAAAGAAAATAAATAGGGAATCA

BPH18   5088   AAACCCAGATTCCAATCGTAGCAACTGTATCATATTAACTCCTTTATATTAGAATCAACA
BPH26   5098   AAACCCAGATTCCAATCGTAGCAACTGTATCATATTAACTCCTTTATATTAGAATCAACA

BPH18   5148   ATTACATTAGTCATAAATGGTTAGCTAATATTATTGTAGCATAATATTTTGAATCCAACC
BPH26   5158   ATTACATTAGTCATAAATGGTTAGCTAATCTTATTGTAGCATAATATTTTGAATCCAACC

BPH18   5208   CAGGATAGGCTTTGCCGGAACTATATTTTGAAACTATGTATAATACATAACCCAAATTAA
BPH26   5218   CAGGATAGGCTTTGCCGGAACTATATTTTGAAACTATGTATAATACATAACCCAAATTAA

BPH18   5268   ACAACAGATAAAGGACGATACACTTCATTTCATGTTTTAAACTTGTATGCTTAAACTTCA
BPH26   5278   ACAACAGATAAAGGACGATGCACTTTATTTCATGTTTTAAACTTGTATGCTTAAACTTCA

BPH18   5328   ATAGTAGAGTTAAAGAAACACCACTTTGGGGTGGGGTGGGGTGCTGTGGGGGCAGCACAA
BPH26   5338   ATAGTAGAGTTAAAGAAACACCACTTGGGGGTGGGGTGGGGTGCTGTGGGGGCAGCACAA

BPH18   5388   GTGGTTTGGTGTTAAAGTCGTGGGTCCCATGATTCTACCTTGCCGATTCACATGTTTCTT
BPH26   5398   GTGGTTTGGTGTTAAAGTCGTGGGTCCCATGATTCTACCTTGCCAATTCACATGTTTCTT

BPH18   5448   CATTAGATGTACAACGGTTGCTTCAGAGGTTTTGGGATTTTTGTTGTTCTTGTTATTTTA
BPH26   5458   CACTAGATGTACAACCGTTGCTTCAGAGGTTTTGGGATTTTTGTTGTTCTTGTTATTTTA

BPH18   5508   TTGTTGTTGTTGATTGCACAATGCGTGAGGATTGGATCATGATCCATGTGGTTCCGAGCG
BPH26   5518   TTGTTGTTGTTGATTGCACGATGCGTGAGGATTGGATCATGATCCATGTGGTTCTGAGCG

BPH18   5568   CACCACAGGCCTTTTGCAAGTGGGTTGTAACTTGTAACTTGGGTACTTACTATTTGAGTG
BPH26   5578   CACCACAAGCCTTTTGCAAGTGGGTTGTAACTTGTAACTTGGGTACTTACTATTTGAGTG

BPH18   5628   GAAAAAAAAT-CAAAATTGTTGAACTAAAACTCTCTATGCAGCTTTAAAATATTTATTTT
BPH26   5638   GAAAAAAAATTCAAAATTGTTGAACTAAAACTCTCTATGCAGCTTTAAAATATTTATTTT
```

BPH18 5687 TATTTTTGATCTTTATGTTGAAATAAATTATAATTTAAAATTTAACCAAAATTTGAAACT
BPH26 5698 TATTTTTGATCTTGATGTTGAAATAAATTATAATTTAAAATTTAACCAAAATTTGAAACT

BPH18 5747 TGATGCAAATTGTAGAACTTTGTACTGAAAATTTTAAAAACTCTAAAAAACAAATCAAAA
BPH26 5758 TTATGCAAATTGTAGAACTTTGTACTGAAAACTTTAAAAACTCTAAAAAACAAATCAAAA

BPH18 5807 CTTCAACTCAAAATAACAAATTTGCTCAGATTTTAAGGCTTTCTACCCAAACTTTATAAC
BPH26 5818 CTTCAACTCAAAATAACAAATTTGCTCAGATTTTAAGGCTTTCTACCCAAACTTTATAAC

BPH18 5867 TTTCAACTCAAATTCTGAAATTTTCAAGTCAAATTTTGTAACTTTCAAGTCATATATTCA
BPH26 5878 TTTCAACTCAAATTCTGAAATTTTCAAGTCAAATTTTGTAACTTTCAAGTCAAATATTCA

BPH18 5927 AAACTACATACCCACAAAAA-CTATATGAAAAATATGATACTCTGAACTCAAAATTCAAA
BPH26 5938 AAACTACATACCCACAAAAAACTATAT-AAAAATATGATACTCTGAACTCAAAATTCAAA

BPH18 5986 ACTTTGTACTCAAATATTTATTTATCATATAGTACAGAAAGTTAGTATCAATGAGAAAGT
BPH26 5997 ACTTTGTACTCAAATATTTATTTATCATATAGTACAGAAAGTTAGTATCAATGAGAAAGT

BPH18 6046 TCAGATGATCTAATTAGATGAAAAAAAAAAACTAGATTAAGCTTGTTGCCCGGGCAGCTA
BPH26 6057 TCAGATGATCTAATTAGATGAAAAAAAAAA-CTAGATTAAGCTTGTTGCCCGGGCAGCTA

BPH18 6106 AGCGCATGGTTGCAGATGTGTGACTTACCTCCTATACAGTACGTGTATTAGCAAACACCT
BPH26 6116 AGCGCATGGTTGCAGATGTGTGACTTACCTCCTATACAGTACGTATATTAGCAAACACCT

BPH18 6166 ACCTACCAAGGTACTCATAGTTATGTATGGTGTGCATGCCTAGTGGTGTGCACGTCTTTT
BPH26 6176 ACCTACCAAGGTACTCATAGTTATGTATGGTGTGCATGCCTAGTGGTGTGCATGTCTTCT

BPH18 6226 ATGTAATTGAAAAAAAGAGACAATATTTTTCTAATTCTCAAATCAAGATGTCATACGGTA
BPH26 6236 ATGCAATTGAAAAAAAGAGACAATATTTTTCTAATTCTCAAATCAAGATGTCATACGGTA

BPH18 6286 AATTACTGCAACAATATGTTCAGAAAAATTCAATCGTAGCCAAACCCTCTGCTGTTCGAT
BPH26 6296 AATTACTGCAACAATATGTTCAGAAAAATTCAATCGTAGCCAAACCCTCTGCTGTTCGAT

BPH18 6346 TTATCACCCTAAATTACAATACCAGACATTTTTAACCTTGAACTTTACAAACCGAACGAA
BPH26 6356 TTATCACCCTAAATTACAATACCAGACATTTTTAACCTTGAACTTTACAAACCGAACGAA

BPH18 6406 TTACCCCCCAAGATGGTTAGAAATTGGTTTTGGTCCTACGTGGCGTATCGGCCCCACAAG
BPH26 6416 TTACCCCCCAAGTTGGTTAGAAATTGGTTTTGGTCCTACGTGGCGCATCGGCCCCACAAG

BPH18 6466 TCAGTCTCCACCTCATCCTCCTCCTATCAGGCTATTGGGGTCCACCTATCAGCTGCTATC
BPH26 6476 TCAGTCTCCACCTCATCCTCCTCCTATCAGGCTATTGGGGTCCACCTATCAGCTGCTATC

BPH18 6526 TCCTTTCTTCAGCTAGAACACAAGAGCAGTCAATGCTCAGGCACTGCCGTTGGACGTCGC
BPH26 6536 TCCTTTCTTCAGCTAGAACACAAGAGCAGTCAATGCTCAGGCACTGCCGTCGGACGTCGC

BPH18 6586 TAGCCTGCTCCCCTTTGTCACCGAGAAAGCCAACTCCACCCCCTCTCTATTCCAGATTGG
BPH26 6596 TAGCCTTCTCCCCTTCGTCACCGAGAAAGCCAACTCCACCCGCTCTCTATTCCAGATTGG

BPH18 6646 ATCTAGACGCTCCCTCCACCACCGATTTGGACTCTGCCGCTACGCCTCCTCCTCTTCTTC
BPH26 6656 ATCTAGACGCTCCCTCCACCACCGATTTGGACTCTGCCGCTACGCCTCCTCCTCTTCTTC

BPH18 6706 TTCCACCGCTGATTAGATCGTCACTGCCTCTTCTGCTCAGGCCGCCGCGATGTGGGCAGG
BPH26 6716 TTCCACCGCTGATTAGATCGTCACTGCCTCTTCTGCTCAGGCCGCCGCGATGTGGGCAGG

BPH18 6766 CAACGCAAAAGGGGCACGGGCAGCTGTGGCAGCACCCCCTTCTTCCCCATCTACAGCCAA
BPH26 6776 CAGCGCAAAAGGGGCGCGGGCAGCTGTGGCAGCACCCCCTTCTTCCCCATCTACAGCCAA

```
BPH18   6826   GGCATTTCAGCAGCAGCAGCTCCACACACATGGTCAACACCGGGGTTCCTCTGCTCCCTC
BPH26   6836   GGCATTTCAGGAGCAGCAGCTCCACACACATGGTCAACACCGGGGTTCCTCTGCTCCCTC

BPH18   6886   GCCACCCCATCGAACACCCGAACCTGTTGCTCCCATATGGCCCCTACGACTCCATGGCTC
BPH26   6896   GCCACCCCATCGAATGCCCGAACCTGTTGCTCCCATATGGCCCCTACGACTCCATGGCTC

BPH18   6946   AACGCGGGCGATAGTGAACTCACGGTAGAGGTGGGACATGTGGCTTCAAACTCACAGTGG
BPH26   6956   AACGCGGGCGACAGTGAACTCACGGCAGAGGTGGGACATGTGGCTTCAAACTCACAGTGG

BPH18   7006   AAGTGGTAAGATCAACGGCGAGGTCCACAACCTCAGCACTGCTCGCCTCCACTATCTCAG
BPH26   7016   AAGTGGTAAGATCAACGGCGAGGTCCACAACCTCAGCACCGCTCGTCTCCACTATCTCAG

BPH18   7066   TCGCGGCGCGTCACTTCTTCTGACCTCAATTCTGCTCTCCTCTGTTAGTTCTGCTGCCTC
BPH26   7076   TCGCGGCGCGTCACTTCTTCTGACCTCAATTCTGCTCTCCTCTGTTAGTTCCGCTGCCTC

BPH18   7126   GTGTCACCCCCAAATCTCAGCGCTGCTTGTCTTGGCTTTGCCTTTAGTGCGCATCTGGTG
BPH26   7136   GTGTCACCCCCAAATCTCAGCGCTGCTTGTCTTGGCTTTGCCTTCAGTGCGCGTCTGGTG

BPH18   7186   GAGATGGTCATCGGCTATAGTAGCCGAGATCGAGCGGGAGAGAGGATGGGGAGAAGAAGA
BPH26   7196   GAGATGGTCATCGGCTATAGTAACCGAGATCGAGCGGGAGAGAGGATGGGGAGAAGAAGA

BPH18   7246   CGGATTGGGCTGACAAATGGGTCCCATCATTCTTTCATTTGTTTTGGCTGAATAGAGTTG
BPH26   7256   CGGATTGGGCTGACAAATGGGTCCCATCATTCTTTCATTTGTTTTGGCTGAATAGAGTTG

BPH18   7306   CCACATGTGTGCCACATAGGATCAAAACCACCTTGGATAGAGTTGGGGGGGGGGGAGGGG
BPH26   7316   CCACATGTGTGCTACAGAGGATCAAAACAGCCTTGGATAGAGTTGGGGGGGG--GAGGGG

BPH18   7366   TAAGTCGTCCGGTTTGTAAAGTTCAGGGTTAAAAAAT-----------------------
BPH26   7374   TAAGTCGTCCGGTTTGTAAAGTTCAGGGTTAAAAAATCTTTTGTATTGTAATTTAGGTGG

BPH18   7402   ------------------------------------TAGGGGTAATTCAGACTTTTTCCT
BPH26   7434   GGGTGGGGGTAAATTAATCGAACGGATGCAAAAGTTTAGGGGTAATTCAGACTTTTTCCT

BPH18   7427   TTATTTTGGCTACACCCGACAAGGAACACCATAAGTGATATGCTGTGAGTGTGTGAGGGA
BPH26   7494   TTATTTTGGCTACACCCGACAAGGAACAC-ATAAGTGATATGTTGTGAGTGTGTGAGGGA

BPH18   7487   GGCATGCATCAGTAATATGTGCAATAAAACTTTATGTAAACTGGCAAATATAGTTCTACT
BPH26   7553   GGCATGCATCAGTAATATGTGCAATAAAACTTTATGCAAACTGGCAAATATAGTTCTACT

BPH18   7547   ATGAAGCAAATAAGTTCCTATATGAGCTGA-CCCCCCCCCCCCACAACCACCATCACACT
BPH26   7613   ATGAAGCAAATAAGTTCCTATATGAGCTGAGCCCCCCCCCCCCACAACCACCATCACAAT

BPH18   7606   AGCTAGTGCAAATCCTCATCATATATATATTGCCAGCTCGAAACTGGCACTCGCTGTCGG
BPH26   7673   AGCTAGTGCAAATCCTCATCATATATATATTGCCAGCTCGAAACTGGCACTCGCTGTCGG

BPH18   7666   TTTGGAACCAACAATTGAAATGGACCGCAGTGATATTTGTTTGAGCAGTACTATCAC-T
BPH26   7733   TTTGGAACCAACAATTGAAATGGACCGCAGTGATATATGTTTGAGCAGTACTATCACAT

BPH18   7725   AGAAAAA------TAGCACTATCC--CGCCAA----------------------------
BPH26   7793   AGGCTAGCTCCAGTAGTGCTGACCGTCACCCACTTCCTTGATGGCTTTTTGTGCGAGCAG

BPH18   7748   ----------GAGTAGAAAAATATATATTT--TTGTAAAAAATCAACACATA--------
BPH26   7853   TGATTCCTCGAGGCAGTCAAGCATCTAGTTGGCCATCCAATGCCCATGCCCACAACCCTT

BPH18   7788   ---------------------------GGCTAGCTCCAGTAGTGCTG-------------
BPH26   7913   GCCATTGCCGTTGCCCGTGCTTGGACCGACTAGATTTGATGGTCCCGGGCCGGCTCATGG
```

```
BPH18  7808  --------------------------------------ACCGTCACCCA-----------
BPH26  7973  CAACCGGATTCATTCTGATCATGCTTCCATTGCCCTTCACCGTCACCAATAGCCCTACTA

BPH18  7819  -------------------------CTTCCTTGATGGCTTTTTGT---------------
BPH26  8033  TGCATTAGGCCTGCCAGATATAACCCTCCTGTTCTGGTCTCTTCTCCATCACCGTCTCGC

BPH18  7839  --------------------------GCGAGCAGTGACTCCTCGAGGC--AGTCAAG---
BPH26  8093  TCGTCGTGCTCAGGTATGGAGGGAGAGCGGATTGGGATCCTCTGACGTGAAGTCAGAGGG

BPH18  7868  ----------CATCATGTGGGCCCCACTGCTCGCGGGCCCACCTGTCAGTGACCACGTCA
BPH26  8153  ATGTGGTGACTGACATGTGGGCCCCACTGCTCACGGGCCCACCTGTCAGTGACCACGTCA

BPH18  7919  CGTGGTAGTTCACGTCAGAGGAACCGCTTCTGAGGGAGAGCAGCGGCGGCGTGCTCGAGA
BPH26  8213  CGTGGTAGTTCACATCAGAGGAGCCGCTTCAGAGGGAGAGCAGCGGCGGCGTGCTCGAGA

BPH18  7979  TAGAGATGTAGACCAAGAGCGAGAGGTTGATCGAGAGACTTAGCATGCTAGTTCATTGGA
BPH26  8273  TAGAGATGTAGACCAAGAACGAGAGGTTGATCGAGAGACTTAGCATGCTAGTTCATTGGA

BPH18  8039  CGTCTCATAGCCCGTTCAGCTTGGCCTTCACTGCTGCTTTCTCTCATGAAATGGTAGTGC
BPH26  8333  CGTCTCATAGCCCGTTCAGCTTGGCCTTCTCTGCTGCTTTCTCTCATGAAATGGTAGTGC

BPH18  8099  AGGGTGAAGATAAAGCTGGCAATATATAGTGTCACTTTCGGGTTTATAAGAAAAACTGAA
BPH26  8393  AGGGTGAAGATAAAGCTGGCAATATATAGTGTCACTTTCGGGTTTATAAGAAAAACTGAA

BPH18  8159  AAAAAA-ACGGTACAGTGATACTTCTGCTACCGGTTTTTCTCATGAACTGATTAATATAT
BPH26  8453  AAAAAAGACGGTATAGTGATACTTCTGCTACCGGTTTTTCTCATGAACTGATTAATATAT

BPH18  8218  GTTATTATTTGAGCATCGGTTTTAGGTTAATCAGCAGCGATTGCGCTGGTTCAGATTGAT
BPH26  8513  GTTATTATTTAAGCATCGGTTTTAGGTTAATCAGCAGCGATTGCGCTGGTTCAGATTGAT

BPH18  8278  GTTTCTATACTAGTGCCAGTTACTCCGATAAATATTTGCAAGAATTTATTTTTTGCACAA
BPH26  8573  GTTTCTATACTAGTGCCAGTTACTCCGATAAATATTTGCAAGAATTTATTTTATGCACAA

BPH18  8338  TTAATTAAAAAAAATAAGTATTTACTACTTAAATTATACACATTTCAGGTATTTAAGGAG
BPH26  8633  TTAATTAAAAAAAATAAGTATTTACTACTTAAATTATACACATTTCAGGTATTTAAGGAG

BPH18  8398  TCAGTAAATTTGGATCAACAAGATCTTGAATTGATCAAAGAAGCGAAACCGATTCTAAAG
BPH26  8693  TCAGTAAATTTGGATCAACAAGATCTTGAATTGATCAAAGAAGCGAAACCGATTCTAAAG

BPH18  8458  AAGTGCAATGGACTTCCCCTTGCAATTGTCACCATAGGTGGTTTCTTGGCAAGCCGCCCC
BPH26  8753  AAGTGCAATGGACTTCCCCTCGCAATTGTCACCATAGGTGGTTTCTTGGCAAGCCGCCCC

BPH18  8518  AAAACTACTTTGGAGTGGAGAAAATTGAATGAGCATATTAGTGCAGAGTTGGAGACAAAC
BPH26  8813  AAAACTACTTTGGAGTGGAGAAAATTGAATGAGCATATTAGTGCAGAGTTGGAGACAAAC

BPH18  8578  CCAGGGCTTGAGGCCATCAGAGCTGTCCTTAATATAAGCTACGACGGATTACCTTATCAC
BPH26  8873  CCAGGGCTTGAGGCCATCAGAGCTGTCCTTAATATAAGCTACGATGGATTACCTTATCAC

BPH18  8638  CTCAAGTCTTGCTTCTTGTATCTGTCCATCTTTCCTGAAGATGGCAAGATTAGCAGAAAA
BPH26  8933  CTCAAGTCTTGCTTCTTGTATCTGTCCATCTTTCCTGAAGATGACAAGATTAGCCGGAAA

BPH18  8698  CGTTTGGTGCGTCGATGGTGTGCAGAGGGTTACTCAAGGGAGCTATGGGACAAATCTGCA
BPH26  8993  CGTTTGGTGCTCCGATGGTGTGCAGAGGGTTACTCAAGGGAGCTATGGGATAAATCTGCA

BPH18  8758  GAGGAAATAGCAAACAACTACTTCTTTGAACTCATAGACAGAAGCATGATCCTACCAACT
BPH26  9053  GAGGAAATAGCAAACAACTACTTCTTTGAACTCATAGACAGAAGCATGATCCTACCAACT
```

```
BPH18   8818   CAAAATTCAACTTACAGCAGTAGAGGGGCTGATTCTTGCCAGATCCATGATATCATGCGT
BPH26   9113   CAAAAATCAACTTACAGCAATAGAGGAGCTGATTCTTGCCAGGTCCACGATATCATGCGC

BPH18   8878   GAGATAGCCATCTTGAAGTCAAAGGAGGAAAACCTTGTTCTTAGACTCGAAGGGGGTCCC
BPH26   9173   GAGATAGCCATCTCGAAGTCAAAGGAGGAAAATCTTGTTCTTAGACTCGAAGGGGGTCGT

BPH18   8938   AGGCTATACAATCATGACACAGTTCGGCATATTTCCATTACAAACAGCAGCGAGGACTGG
BPH26   9233   AGGCTACACAATCATGACATAGTTCGACATCTTTCCATTACAAACAGCAGCGAGGACTGG

BPH18   8998   GAGACAGATGTCGGTGAATTGAAGACAACAGTAGACATGTCCCGAATAAGATCATTAACA
BPH26   9293   GAGACAGATGTCGGTGAATTGAAGACAACAGTAGACATGTCCCGAATAAGATCATTAACA

BPH18   9058   GTATTTGGGATGTGGAGACCTTTTTTTATTTCTGACAAGATGCAGTTACTACAAGTGCTA
BPH26   9353   GTGTTTGGGGAGTGGAGGCCAATTTTCATTTCTGACAAGATGAGGTTGTTGCGTGTGCTC

BPH18   9118   GACTTGGAAGACACAAACGGTGTATATGATCATCATATTAAGCAAATTGGGAAGCTCCTT
BPH26   9413   GACTTGGAAGACACAGAAGGTGTACGTAATCACCATATTAAGCAAATAGGGGAGCTTCTT

BPH18   9178   CACCTTAGATACCTTTCTCTAAGAGGATGTAGGGACATTACTTACCTGCCTGATTCCTTA
BPH26   9473   CACCTTAGATACCTTTCTCTAAGAGGATGTACGCACATTGCTTACATGCCTGATTCTTTG

BPH18   9238   GGTAACCTAAGGCAACTGGAGACACTAGATGTCAGAGGTACGTGCATACTCAGGTTGCAA
BPH26   9533   GGTAACCTAAGGCAACTGGAGACACTAGATGTCAGAGATACGTTCATACTCAGGTTGCCA

BPH18   9298   AAGACCATCATTAATCTTCGCAAGCTAAAGTATCTCCGTGCTGTCCCAAATTTATCTGAC
BPH26   9593   AAGACCATCACTAATCTTTGCAAGCTGAAGTATCTCCGTGCAAGTAAAGACCT---CAAC

BPH18   9358   CCGTATGAAGACATAGCAGAGAAACTACCAGAGCTCATTAGGAACAGGCTATGCATTTCT
BPH26   9650   TTCTATGAAGGCATAAGAGAGAAACTACCAGAGCTCATGAGGAACAGGCTATGCATTTTT

BPH18   9418   GCGACTGTGTTGCTGGCGCTTTGCGTGTTATGCTCAGCAAGTGCAATATGTAAGTTTGAT
BPH26   9710   ACCGCTGCGTTGCTGGGGCTTTGTCTGGCATGCTCAGCTAGTGCGATTGGTAAGTTTGAT

BPH18   9478   GTAGGGATTAGTACCCGTGACCTCTGCACCTTGTGTTGCTGCAGTATTCTCCCTGCCATT
BPH26   9770   GAAGAGATTAATACCCGTGATGTCTGCACCATGTGTTGCTGCAGTATTCTCCCTAGCATT

BPH18   9538   GCCATGCGCCTCGACGGGAATGGTGTAGTAGCACCGAGAGGGCTGAGGA------GACTG
BPH26   9830   GCTATGCGCCTCCAAGGGAATGGTGTAGTAGCACCAAGAGGGCTGAGGATCAGGAGACTG

BPH18   9592   ACAGCCCTGCACACGCTAGGTGTGGTGGACATTTCATGGCAGCCATCAATTTTACAAGAT
BPH26   9890   ACAGCCCTGCACACGCTAGGTGTGGTGGACATTTCATGGCAGCCATCAATTTTACAAGAT

BPH18   9652   ATCAAGAGGCTCATCCAGCTGCGCAAACTGGGAGTGAGCGGTGTCAACAAGAAAAACAGC
BPH26   9950   ATCAAGAGGCTCATCCAGCTGCGCAAATTGGAAGTGACCGGTGTCAACAAGAAAAATAGC

BPH18   9712   AAAAAGTTTTTATCTGCCCTTGTCGCTCTCAGCCGCCTGGAATCATTGTCACTGATCTCG
BPH26   10010  AAAAAGCTTTTCTCTGCCCTTGCCGCTCTCAGCCAACTAGAATCATTGTCACTGTTCTCG

BPH18   9772   AAGGGGAAGCCAGGTCTCTGGGGCTGTCTGGATGCTGATGAAAAGTTTTCGCCACCTAAG
BPH26   10070  AAGTGGAAGCCAGGTCTCTGGGGCTGTCTGGATGCTGAAGAAAAGTTTTCGCCACCTAAG

BPH18   9832   AATCTCAAGACTCTGAAGCTTCAAGGCAACCTGGTTGGGTTGCCAAAATGGATCGGGCAG
BPH26   10130  AATCTCAAGACTCTGAAGCTTCAAGGCAACCTGGTTGAGCTGCCAAAATGGATCGGGAAG

BPH18   9892   CTCAACAATCTCGTGAAGCTGAAGCTATCAGAAACCGGGCTCAAGGATCATGATGCTGCT
BPH26   10190  CTCAACAATCTTGTTAAGCTGAAGCTATCAAAATCCAGGCTCAAGGATCATGATGCTGCT
```

```
BPH18   9952   ATACAAGTCCTTGGTAAGCTACGAAACCTGACCATCCTATGCCTGCTGGGCAAGTCATTT
BPH26   10250  ATACAAGTCCTTGGTATGCTACCAAACCTGACCATCCTATGCCTGCCGCGCAAGTCATTT

BPH18   10012  CACTCGCTTGAGGGTGGTGAACTCAATTTCTCGGAGGGATCTTTCAAAAGCCTGGTGGTT
BPH26   10310  CACTCGCTTGAGGGTGGCGAACTCAATTTCTCGGAAGGATCTTTCAAAAGCCTGTTGGTT

BPH18   10072  CTCGAGCTTGACTTGAGTGGGAGCAAATGCGTCAAGTTTCAACAAGGAGCATTCCACAAT
BPH26   10370  CTCGAGCTTGACTTCAGTGGGAGCAAAGGTGTTACGTTTCAACAAGGAGCATTCCGCAAA

BPH18   10132  CTTGAGCTACTGGAGCTTGATTGTGAGCCACGGGAGCTTTCTGCAGGTCATATTGAAGAA
BPH26   10430  CTAGAGCTGTT--------ATTG-------------CTTTC---AGTTCATAGTGAAGAA

BPH18   10192  ATCAAAACTAAGTTCTCTGGGCTAGAATTTCTCCCAAGAATCAAGGAAGTCCGGCTCCAG
BPH26   10466  GTTCAAAGTAAATTATCTGGACTAGAATTTCTCCAAAGCATCAAGGAAGTTCAGATCGAT

BPH18   10252  GGTTATTTTTACGGATCTTGTGACACACGAAAATTGAAGGAGGACTTGCTGGCACAGCTT
BPH26   10526  GGTTATTGCCCA---------AACGAGGAAGGATTGAAGAAAGACTTGCTGGTCCAACTT

BPH18   10312  TCCGAGAACCCAAAGAAACCAATCCTGAAGACTAGCAGGTGA------
BPH26   10577  TCTCAAAATCCAAAGAAACCCTTTCTGAAGATCGACGGTTATTTTTAA
```

Supplementary Figure S10. Genomic sequence alignment between *BPH18* and *BPH26*. The sequences of BPH18 and BPH26 are from their donors, IR65482 and ADR52. Translate start codon and stop codon were boxed and protein coding sequences were underlined. Yellow, pink, and green indicate identical nucleotide, different nucleotide, and *indel* nucleotide, respectively.

```
IR24    1    MEATAVSIGRSVLKGALGFAKSTLVEEVSLQLGVQRDQAFIRDELEMMNSFLMAANDEKD
BPH26   1    MEATAVSIGRSVLKGALGFAKSTLVEEVSLQLGVQRDQAFIRDELEMMNSFLMAANDEKD
BPH18   1    MEATAVSIGRSVLKGALGFAKSTLVEEVSLQLGVQRDQAFIRDELEMMNSFLMAANDEKD

IR24    61   DNKVVRTWVKQVRDVAYDVEDCLQDFAVRLGRKSSSWWLSPHTLWERRRIAKQMEELRGK
BPH26   61   DNKVVRTWVKQVRDVAYDVEDCLQDFAVRLGGKSSTWWLSPHTLWERRRIAKQMKELRGK
BPH18   61   DNKVVRTWVKQVRDVAYDVEDCLQDFAVRLGGKSSTWWLSPHTLWERRRIAKQMKELRGK

IR24    121  VEDVSQRNMRYQLIKGSKPTVATNVAPSSTARATMSGAHEERWQHEKAIDHLVRLVKTKV
BPH26   121  VEDVSQRNMRYQLIKGSKPTVATNVTPSSTARATMSGAHEERWQHEKAIDHLVRLVKTKV
BPH18   121  VEDVSQRNMRYQLIKDSKPTVATNVTPSSTARATMSGAQEERWQHEKAIDHLVRLVKTKV

IR24    181  DERRVIAVWGTSGDIREMSIVGGAYDHLKRSNKFECCAWVNLMHPLNPTKLLQTIVRQFY
BPH26   181  DERRVIAVWGTSGDIREMSIVGGAYDHLKRNNKFECYAWVNLMHPLNPTKLLQTIVRQFY
BPH18   181  DERRVIAVWGTSGDIREMSIVGGAYDHLKRNNKFECCAWVNLMHPLNPTKLLQTIVRQFY

IR24    241  VRSLQEAGKATPSCQILSSMLIKEDQGLGFRVLRSMLMKEDHLNDEFNKYLSDKCYLIVL
BPH26   241  VRSLQEAGKATPSCQILSSMLIKEDQGLGFRVLRSMLMKEDHLNDEFNKYLSDKCYLIVL
BPH18   241  VRSLQEAGKATPSCQILSSMLIKEDQGLGFRVLRSMLMKEDHLNDEFNKYLSDKCYLIVL

IR24    301  NDLSSAEEWKQIKMLFPDNKKGSRIIVFTQQVEVASFCARTEEVAPEHMQLFADQTLYAF
BPH26   301  NDLSTAEEWKQIKMLFPDNKKGSRIIVFTQQVEVASFCARTEEVAPEHMQLFADQTLYAF
BPH18   301  NDLSTAEEWKQIKMLFPDNKKGSRIIVFTQQVEVASFCARTEEVAPEHMQLFADQTLYAF

IR24    361  HCKGAKDGVDSMEDSSNLNEDTTYNAVEGKSLTRTYSMVTAFKESEIVGRVDEIKEIIEL
BPH26   361  HCKGAKDGVDSMEDSSNLNEDTTYNAVEGKSLTRTYSMVTAFKESEIVGRVDEIKEIIEL
BPH18   361  HCKGAKDGVDSMEDSSNLNEDTTYNAVEGKSLTCTYSMVTAFKESEIVGRVDEIKEIIEL

IR24    421  ISKGSQQLEKISVWGMGGIGKTTLIQNVYRSEKVKKMFDKHACVTIMRPFNLDDLLKSLV
BPH26   421  ISKGSQQLEKISVWGMGGIGKTTLIQNVYRSEKVKKMFDKHACVTIMRPFNLNDLLMSLV
BPH18   421  ISKGSQQLEKISVWGMGGIGKTTLIQNVYRSEKVKKMFDKHACVTIMRPFNLNDLLMSLV

IR24    481  RQLEDSKTSGEKELTSILEEKKYLIVLDDVLSTTEWNAIESYFPAMETGSRIIITTRHES
BPH26   481  RQLEDSKTSGGKELVSILEGKKYLIVLDDVLFTTEWDAIESYFPATETGSRIIITTRHES
BPH18   481  RQLEDSKTSGGKELVSILEGKKYLIVLDDVLFTTEWDAIESYFPATETGSRIIITTRHES

IR24    541  IAKHCSGDQQGKIYQLNRLGDNDAKNLFAKKVFKEPVNLDQEDLELIEEAKLILKKCKGL
BPH26   541  IAKHCSGDQQGKMYQLNRLGDNDAKNLFAKKVFKESVNLDQQDLELIKEAKPILKKCNGL
BPH18   541  IAKHCSGDQQGKMYQLNRLGDNDAKNLFAKKVFKESVNLDQQDLELIKEAKPILKKCNGL

IR24    601  PLAIVTIGGFLASRPKTALEWRKLNEHISAELETNPELEAIRAVLNISYDGLPYHLKSCF
BPH26   601  PLAIVTIGGFLASRPKTTLEWRKLNEHISAELETNPGLEAIRAVLNISYDGLPYHLKSCF
BPH18   601  PLAIVTIGGFLASRPKTTLEWRKLNEHISAELETNPGLEAIRAVLNISYDGLPYHLKSCF

IR24    661  LYLSIFPEDDKISRKRLVRRWCAEGYSRELLDKSAEEIANNYFFELIDRSMIILPTQKST
BPH26   661  LYLSIFPEDDKISRKRLVLRWCAEGYSRELWDKSAEEIANNYFFELIDRS-MILPTQKST
BPH18   661  LYLSIFPEDGKISRKRLVRRWCAEGYSRELWDKSAEEIANNYFFELIDRS-MILPTQNST

IR24    721  YSNRGADSCQVHDIMREIAISKSKEENLVLRLEGGRRLHNHGTVRHLSITNSNEDWETDV
BPH26   720  YSNRGADSCQVHDIMREIAISKSKEENLVLRLEGGRRLHNHDIVRHLSITNSSEDWETDV
BPH18   720  YSSRGADSCQIHDIMREIAILKSKEENLVLRLEGGPRLYNHDTVRHISITNSSEDWETDV

IR24    781  GELKRTVDMSRIRSLTVFGEWRPFFISDKMRLLRVLDLEDTEGVRNHHIKQIGELLHLRY
BPH26   780  GELKTTVDMSRIRSLTVFGEWRPIFISDKMRLLRVLDLEDTEGVRNHHIKQIGELLHLRY
BPH18   780  GELKTTVDMSRIRSLTVFGMWRPFFISDKMQLLQVLDLEDTNGVYDHHIKQIGKLLHLRY
```

```
IR24    841  LSLRGCIDIAYLPDSLGNLRQLETLDIRDTFILRLPKTITNLRKLKYLRASIDLM-IVEV
BPH26   840  LSLRGCTHIAYMPDSLGNLRQLETLDVRDTFILRLPKTITNLCKLKYLRASKDLN-FYEG
BPH18   840  LSLRGCRDITYLPDSLGNLRQLETLDVRGTCILRLQKTIINLRKLKYLRAVPNLSDPYED

IR24    900  IIEKLPELMRNRLCIFTAALLLFCLACTTSSIGMLNGGINTRDVCTLGCCSFLPSIAMLL
BPH26   899  IREKLPELMRNRLCIFTAALLGLCLACSASAIGKFDEEINTRDVCTMCCCSILPSIAMRL
BPH18   900  IAEKLPELIRNRLCISATVLLALCVLCSASAICKFDVGISTRDLCTLCCCSILPAIAMRL

IR24    960  DGSGVVAPRGL--RRLTALHTLGVVDISWESSVLQDLKKLTQLRKLEVTGVNKKNSKKFF
BPH26   959  QGNGVVAPRGLRIRRLTALHTLGVVDISWQPSILQDIKRLIQLRKLEVTGVNKKNSKKLF
BPH18   960  DGNGVVAPRGL--RRLTALHTLGVVDISWQPSILQDIKRLIQLRKLGVSGVNKKNSKKFL

IR24    1018 SALAALSRLESLSLLSKGKPGLCGCLDAEEKFSPPKDLKSLKLQGNLVELPKWIRQLNNL
BPH26   1019 SALAALSQLESLSLFSKWKPGLWGCLDAEEKFSPPKNLKTLKLQGNLVELPKWIGKLNNL
BPH18   1018 SALVALSRLESLSLISKGKPGLWGCLDADEKFSPPKNLKTLKLQGNLVGLPKWIGQLNNL

IR24    1078 VKLKLSETMLKDHDAAIQVLGMLPNLTILCLSRESFHSLEGGELNFSEGSFKSLVVLKLH
BPH26   1079 VKLKLSKSRLKDHDAAIQVLGMLPNLTILCLPRKSFHSLEGGELNFSEGSFKSLLVLELD
BPH18   1078 VKLKLSETGLKDHDAAIQVLGKLRNLTILCLLGKSFHSLEGGELNFSEGSFKSLVVLELD

IR24    1138 FGGSKCVKFQQGAFHDLELLLLSVYS--------EEVETKFSGLEFLQSIKEVQIDGYYP
BPH26   1139 FSGSKGVTFQQGAFRKLELLLLSVHS--------EEVQSKLSGLEFLQSIKEVQIDGYCP
BPH18   1138 LSGSKCVKFQQGAFHNLELLELDCEPRELSAGHIEEIKTKFSGLEFLPRIKEVRLQGYFY

IR24    1189 ---NWKGLKKDLLVQLSQNPKKPFLKTGRNF
BPH26   1190 ---NEEGLKKDLLVQLSQNPKKPFLKIDGYF
BPH18   1198 GSCDTRKLKEDLLAQLSENPKKPILKTSR--
```

Supplementary Figure S11. Amino acid sequences alignment of BPH18/BPH26 protein among IR24, IR65482 (*BPH18* donor), and ADR52 (*BPH26* donor). Identical amino acid, similar amino acid, different amino acid, and gap were represented with yellow, green, pink, and gray, respectively. The leucine-rich repeat (LRR) domain is underlined.

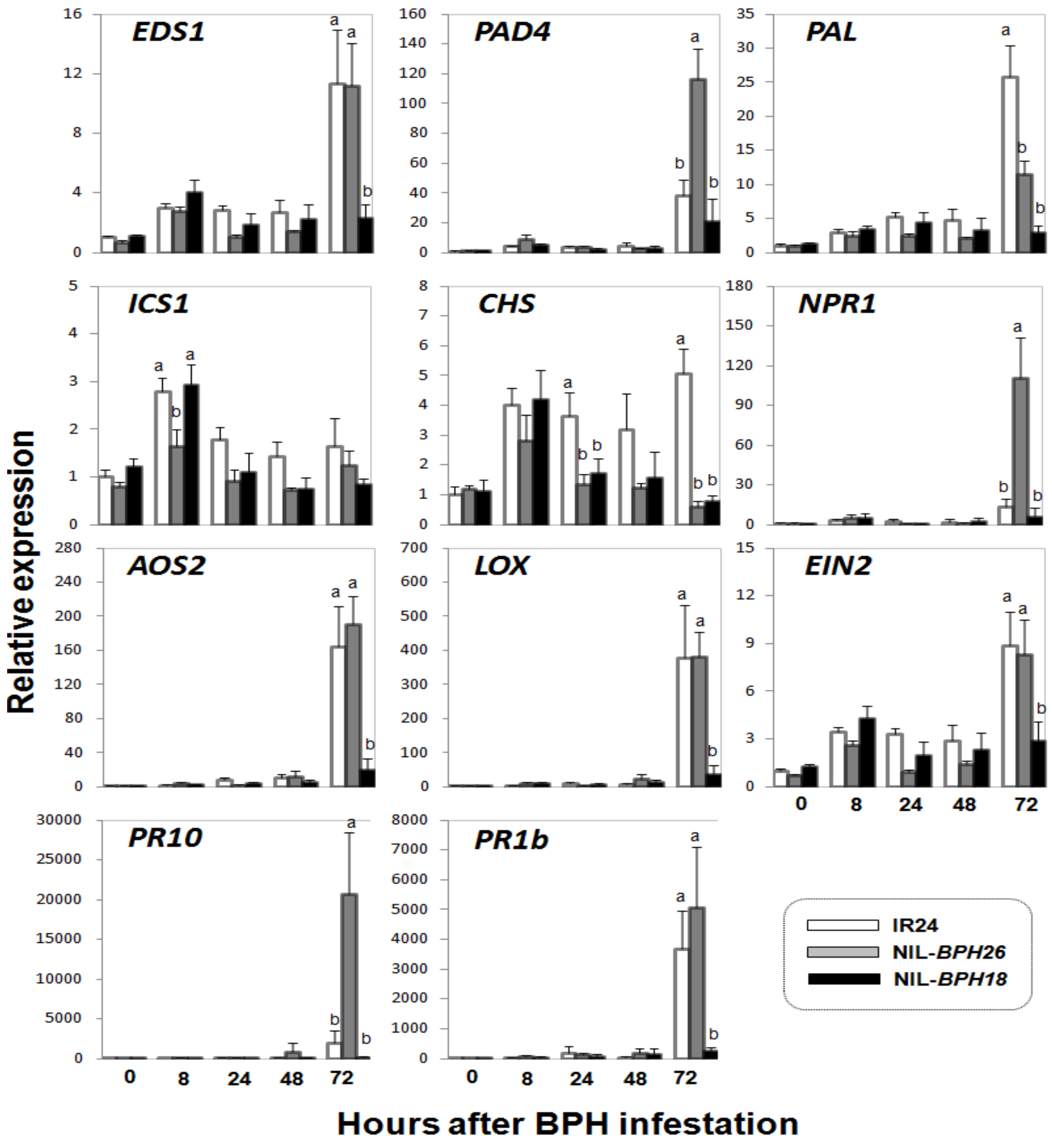

Supplementary Figure S12. Expression analysis of plant defense-related genes in IR24, NIL-*BPH18*, and NIL-*BPH26* after BPH infestation using qRT-PCR. *EDS1*, *PAD4*, *PAL*, *ICS1*, and *CHS* are the SA synthesis-related genes. *NPR1* is a key regulator of SA-dependent systemic acquired resistance. *AOS2* and *LOX* are involved in JA-synthesis pathway. *EIN2* is the ethylene receptor gene. *PR1b* and *PR10* are the pathogen-related genes. *OsAct1* gene was used as an internal control for the calculation of relative expression value. The value of 0 h from IR24 was set as 1 in relative expression value. Statistical difference shown as *a* or *b* was obtained by Duncan's multiple range test (α=0.05).

Supplementary Table S1. List of the markers used in fine-mapping

Marker	Forward primer sequence (5'->3')	Reverse primer sequence (5'->3')	Restriction Enzyme	Marker type
BN52	GGAATCAAATCATTGACCACACG	CGCTTTCGTCTCTCCTGACTGTAT	PstI	CAPS
BN8	GCCTGATTTTGTTGCATGCA	CTGATGAGCAATTTAGGCATTT	SacI	CAPS
BN88	CTGACGCCGCCCCTCCTACT	GGGTGTACTGCGGTGTTGCCT	StuI	CAPS
pBPH9	AGCGCTGGTCGTTGGGGTTGTAGT	ATTAAAAGTGATCGCAGCCGTTCG	-	Indel
BIM3	TCTCCCTAGCATTGCTATGC	TCCTGGGAGAAATTCTAGCC	HindIII	CAPS
7312T4	ACGGCGGTGAGCATTGG	TACAGCGAAAAGCATAAAGAGTC	HinfI	CAPS
BN162	TCAGCATGAATTACTCGCGGTTA	TTTGTTTGTTTGGAAGCACTCCG	PstI	CAPS
BN123	CCTTATTTTCGAGTGGAATTTGTGG	CGCTTTTCAAACGGTTAAACGT	EcoRV	CAPS
BN72	GAGGAATATCGACCACTTGACCC	GGAAGATCAAGTTCAAGGCGTTTG	EcoRV	CAPS
BN45	CTGGAACGAGAGCTTCATACAT	CTCACCTCTGGGACCTAACTTC	XhoI	CAPS

Supplementary Table S2. The primers used for molecular analysis of the *BPH18* gene

Primer name	Sequence (5'->3')
NF	ACGGCGGTGAGCATTGG
NR	AGCTCCCTTGAGTAACCCTCTGCAC
NFCF	ACTCATCCATCCATCACACAAGTTACTAGCAATCCACG
NFCR	CAGGAAAATATTCCATAGAACAGAAAATTATGTTG
RPL2F	CGGGTGCCGATCAATAGTTTTCTTCGAATTTACTG
RPL2R	TGTGTCTGATGGTAAAGAGGTTTCCAATGGACAAA
LRR-8.0-F	GACTCATACTCTAGGCCTCCTTCTGGGTTATGATCCCATCCTATAATA
LRR-8.0-R	TCATAACCCAGAAGGCCTGGTTTCGGTTTCGGGTTGTTTGTTTGGATATCCG
BPH18-pro-F	TCTAGAGATGTAGCCTCCTTTTGCAG
BPH18-pro-R	TCTAGAGGCTTTGCTCTCCTCTCCTCG
BPH18-ORF-F	TCTAGAATGGAGGCCACGGCGGTGAGC
BPH18-ORF-R	GGATCCTCACCTGCTAGTCTTCAGGATTG
BPH18-ter-F	GGATCCTTTTAGCTGATCGAGCTCCATCGCAAAT
BPH18-ter-R	CCCGGGTTGTTGGTGATGCTATTAGTTCGC
BPH18i-F	AAAAAGCAGGCTGCGGTGAGCATTGGCAGGTC
BPH18i-R	AGAAAGCTGGGTGCTGCTGGGTGTGACATTGGTAG
qPCR-BPH18-F	TGCTGATCAGACTCTTTATGCTTTTC
qPCR-BPH18-R	AGTGTCTTCGTTTAAGTTAGATGAGTCCT
qPCR-BPH18-probe	AGGGTGCTAAAGATGGAGTAG
qPCR-Ubiquitin-F	CCGCCACTACTGCGGTAAGT
qPCR-Ubiquitin-R	ACGCCTAAGCCTGCTGGTT
qPCR-Ubiquitin-probe	CGGCCTCACCTACG
attB-BPH18-pro-F	<u>GGGGACAAGTTTGTACAAAAAAGCAGGCT</u>CGGGTGCCGATCAATAGTTT
attB-BPH18-pro-R	<u>GGGGACCACTTTGTACAAGAAAGCTGGGT</u>GGCTTTGCTCTCCTCTCCTCG
BPH18-loc-F	<u>CCCGGG</u>ACTCATCCATCCATCACACAAG
BPH18-loc-R	A<u>ACTAGT</u>CCTGCTAGTCTTCAGGATTG
BPH18-ind2-F	TGGGCTGACAAATGGGTCC
BPH18-ind2-R	CCTTGTCGGGTGTAGCCAA

Underlined and double underlined sequences are *attB* sites for Gateway® cloning and restriction enzyme sites for cloning, respectively.
